# Supplementary material for: Malaria awareness of adults in high, moderate and low transmission settings: A cross-sectional study in rural East Nusa Tenggara Province, Indonesia
Source: PLoS One. 2021 Nov 15;16(11):e0259950. doi: 10.1371/journal.pone.0259950 (PMC8592438; doi:10.1371/journal.pone.0259950)
Supplement: S1 Dataset — (PDF) [file pone.0259950.s002.pdf]

#	Res_ID	MES	Sex	Edu	AG	SES	Q1	Q2	Q3	Q4	Q5	Q6	Q7	Q8	Q9	Q10	K_1	K_2	MKS	MA	ABUM	ABMK
1	10111	3	1	3	4	3	1	1	1	1	1	1	0	1	0	1	1	1	4	1	1	1
2	10112	3	1	5	2	3	1	1	1	1	1	0	1	0	0	1	1	0	3	1	1	1
3	10113	3	1	2	3	2	1	1	1	1	1	0	1	0	0	1	1	0	3	1	1	1
4	10114	3	1	5	1	2	1	1	1	1	1	0	1	1	1	1	1	1	4	1	1	1
5	10115	3	1	3	4	2	1	1	1	0	1	1	0	0	0	1	1	0	3	1	1	0
6	10116	3	1	3	3	2	1	1	1	1	1	0	0	1	0	1	1	0	3	1	1	1
7	10117	3	1	5	1	2	1	1	1	1	1	0	1	1	1	1	1	1	4	1	1	1
8	10118	3	1	4	3	2	1	1	1	1	1	0	0	1	1	1	1	1	4	1	1	1
9	10119	3	1	3	3	2	1	1	1	1	1	0	0	0	1	1	1	0	3	1	1	1
10	101110	3	1	2	4	2	1	0	1	1	1	0	0	1	0	1	1	0	3	1	1	1
11	101111	3	1	2	3	2	1	0	1	1	1	0	1	0	0	1	1	0	3	1	1	1
12	101112	3	1	5	1	2	1	1	1	1	1	0	1	1	0	1	1	1	4	1	1	1
13	101113	3	1	4	4	2	1	0	1	1	1	0	0	1	0	1	1	0	3	1	1	1
14	101114	3	1	5	5	3	1	1	1	1	1	0	0	1	0	1	1	0	3	1	1	1
15	101115	3	0	1	5	1	0	0	0	0	0	0	0	0	0	1	0	0	2	0	0	0
16	101116	3	0	3	2	2	1	1	1	0	1	0	0	1	0	1	1	0	3	1	1	0
17	101117	3	1	5	3	3	1	1	1	1	1	1	0	1	0	0	1	1	3	1	1	1
18	101118	3	1	3	4	2	1	1	1	1	1	0	0	1	1	1	1	1	4	1	1	1
19	101119	3	1	3	2	2	1	1	1	0	1	0	0	0	1	1	1	0	3	1	1	0
20	101120	3	0	2	4	1	1	0	1	0	1	0	0	0	0	1	0	0	2	0	1	0
21	101121	3	1	3	3	1	1	1	1	0	1	0	0	0	0	1	0	0	2	0	1	0
22	101122	3	0	1	4	1	1	1	1	0	1	1	0	0	0	1	1	0	3	1	1	0
23	101123	3	0	3	4	2	1	1	1	1	1	0	1	0	0	1	1	0	3	1	1	1
24	101124	3	0	1	4	2	1	1	1	1	1	1	0	0	0	1	1	0	3	1	1	1
25	101125	3	1	1	5	2	1	1	1	0	0	0	0	1	0	1	1	0	2	0	1	0
26	101126	3	0	3	3	2	1	1	1	1	1	0	0	1	0	1	1	0	3	1	1	1
27	101127	3	1	3	3	2	1	1	1	1	1	1	0	0	0	1	1	0	3	1	1	1
28	101128	3	1	3	4	2	1	1	1	1	1	0	1	0	0	1	1	0	3	1	1	1
29	101129	3	0	4	2	1	1	0	1	1	1	0	0	1	0	1	1	0	3	1	1	1
30	101130	3	1	3	4	2	1	1	1	1	1	0	0	0	1	1	1	0	3	1	1	1
31	10121	3	0	3	1	2	1	1	1	1	1	0	1	0	1	1	1	1	4	1	1	1
32	10122	3	1	4	2	2	1	1	1	1	1	0	1	1	1	1	1	1	4	1	1	1
33	10123	3	0	2	2	2	1	1	1	1	1	0	1	1	1	1	1	1	4	1	1	1
34	10124	3	0	4	1	2	1	1	1	1	1	0	1	0	1	1	1	1	4	1	1	1
35	10125	3	0	4	1	2	1	0	1	0	1	0	1	0	0	1	1	0	2	0	1	0
36	10126	3	0	1	5	1	0	0	0	0	0	0	0	0	0	1	0	0	2	0	0	0
37	10127	3	1	1	5	1	1	0	1	1	1	0	1	1	1	0	1	1	3	1	1	1
38	10128	3	0	2	3	2	1	1	1	1	1	0	1	0	1	1	1	1	4	1	1	1
39	10129	3	1	1	3	2	1	1	1	1	1	0	1	0	1	0	1	1	3	1	1	1
40	101210	3	0	1	2	2	1	1	1	1	1	0	1	0	0	1	1	0	3	1	1	1
41	101211	3	0	1	4	2	1	1	1	1	1	1	1	0	1	1	1	1	4	1	1	1
42	101212	3	0	4	2	3	1	1	1	1	1	1	0	1	1	0	1	1	4	1	1	1
43	101213	3	1	2	5	2	1	1	1	1	1	1	0	1	0	0	1	1	3	1	1	1
44	101214	3	1	1	4	2	1	1	1	0	1	1	0	1	1	1	1	1	4	1	1	1
45	101215	3	0	1	1	2	0	0	0	0	0	0	0	0	0	0	0	0	1	0	0	0
46	101216	3	0	1	5	1	1	1	1	0	1	1	1	0	0	1	1	1	3	1	1	1
47	101217	3	1	2	2	2	1	1	1	1	1	1	0	1	0	1	1	1	4	1	1	1
48	101218	3	0	2	1	2	1	0	1	1	1	0	1	0	1	1	1	1	3	1	1	1
49	101219	3	1	3	2	2	1	1	1	1	1	0	0	1	1	1	1	1	4	1	1	1
50	101220	3	0	2	2	2	1	1	1	1	1	1	0	1	1	1	1	1	4	1	1	1
51	101221	3	1	3	2	2	1	1	1	1	1	0	0	1	1	1	1	1	4	1	1	1
52	101222	3	1	4	2	3	1	1	1	1	1	1	1	1	1	1	1	1	4	1	1	1
53	101223	3	1	1	4	2	1	1	1	1	1	0	1	1	1	1	1	1	4	1	1	1
54	101224	3	0	2	3	2	1	1	1	1	1	1	0	1	1	1	1	1	4	1	1	1
55	101225	3	1	3	1	2	1	1	1	1	1	0	1	1	1	1	1	1	4	1	1	1
56	101226	3	0	2	3	1	1	1	1	1	1	1	0	1	0	0	1	1	3	1	1	1
57	101227	3	1	3	2	2	1	1	1	1	1	1	0	1	1	1	1	1	4	1	1	1
58	101228	3	0	3	3	2	1	1	1	1	1	1	0	0	0	1	1	0	3	1	1	1
59	101229	3	1	4	2	2	1	1	1	1	1	1	1	1	1	1	1	1	4	1	1	1

60	101230	3	1	3	3	3	1	1	1	1	1	0	0	1	0	1	1	0	3	1	1	1
61	101231	3	1	1	5	1	0	0	0	0	0	0	0	0	0	1	0	0	2	0	0	0
62	101232	3	1	1	5	1	1	1	1	1	1	0	1	1	0	1	1	1	4	1	1	1
63	101233	3	1	1	2	1	1	1	1	1	1	0	0	1	0	1	1	0	3	1	1	1
64	101234	3	1	2	3	1	1	1	1	0	1	0	1	0	0	1	1	0	3	1	1	0
65	101235	3	1	1	5	1	1	1	1	1	1	0	1	0	0	1	1	0	3	1	1	1
66	10131	3	0	3	3	2	1	1	1	1	1	0	0	1	0	1	1	0	3	1	1	1
67	10132	3	1	4	1	2	1	1	1	1	1	0	1	0	0	1	1	0	3	1	1	1
68	10133	3	1	4	3	2	1	1	1	1	1	0	1	0	0	1	1	0	3	1	1	1
69	10134	3	0	4	2	2	1	1	1	1	1	0	0	1	0	1	1	0	3	1	1	1
70	10135	3	0	4	1	2	1	0	1	0	1	0	1	0	0	1	1	0	2	0	1	0
71	10136	3	0	4	3	2	1	1	1	1	1	0	1	0	0	1	1	0	3	1	1	1
72	10137	3	1	4	2	3	1	1	1	1	1	1	0	0	0	1	1	0	3	1	1	1
73	10138	3	0	4	2	2	1	1	1	1	1	0	0	1	0	1	1	0	3	1	1	1
74	10139	3	0	3	2	2	1	1	1	1	1	0	0	1	0	1	1	0	3	1	1	1
75	101310	3	1	4	2	2	1	1	1	1	1	0	0	1	0	1	1	0	3	1	1	1
76	101311	3	1	4	1	2	1	1	1	1	1	0	0	1	0	1	1	0	3	1	1	1
77	101312	3	0	4	2	2	1	1	1	1	1	1	0	0	0	1	1	0	3	1	1	1
78	101313	3	0	1	3	1	1	1	1	0	1	1	0	0	0	1	1	0	3	1	1	0
79	101314	3	1	4	2	2	1	1	1	1	1	0	0	1	0	1	1	0	3	1	1	1
80	101315	3	1	3	1	1	1	1	1	0	1	0	0	1	0	1	1	0	3	1	1	0
81	101316	3	0	3	3	2	1	1	1	1	1	1	0	1	0	1	1	1	4	1	1	1
82	101317	3	0	3	2	2	1	1	1	1	1	1	0	1	0	1	1	1	4	1	1	1
83	101318	3	0	4	4	2	1	1	1	1	1	0	0	1	0	1	1	0	3	1	1	1
84	101319	3	1	3	3	3	1	1	1	1	1	0	0	1	0	1	1	0	3	1	1	1
85	101320	3	1	2	5	2	1	1	1	1	1	0	0	1	0	1	1	0	3	1	1	1
86	101321	3	0	2	4	2	1	1	1	1	1	0	0	1	0	1	1	0	3	1	1	1
87	101322	3	1	3	2	2	1	1	1	1	1	0	0	1	0	1	1	0	3	1	1	1
88	101323	3	0	1	2	2	1	1	1	1	1	0	0	1	0	1	1	0	3	1	1	1
89	101324	3	1	2	4	2	1	1	1	1	1	0	0	1	0	1	1	0	3	1	1	1
90	101325	3	1	3	3	2	1	1	1	1	1	1	0	0	0	1	1	0	3	1	1	1
91	101326	3	0	1	1	2	1	1	1	1	1	0	0	1	0	1	1	0	3	1	1	1
92	101327	3	1	3	1	2	1	1	1	1	1	0	0	1	0	1	1	0	3	1	1	1
93	101328	3	1	1	5	2	1	1	1	1	1	0	0	1	0	1	1	0	3	1	1	1
94	101329	3	0	4	1	2	1	1	1	1	1	0	0	1	0	1	1	0	3	1	1	1
95	101330	3	1	3	5	2	1	1	1	1	1	1	0	0	0	1	1	0	3	1	1	1
96	10141	3	1	5	2	3	1	1	1	0	0	0	1	1	0	1	1	1	3	1	1	0
97	10142	3	0	4	2	2	1	1	1	1	0	0	1	0	0	0	1	0	2	0	1	0
98	10143	3	1	2	5	2	1	1	1	0	1	0	1	1	0	0	1	1	3	1	1	0
99	10144	3	0	4	1	2	1	1	1	0	0	0	1	0	0	0	1	0	2	0	1	0
100	10145	3	1	1	4	1	0	0	0	0	0	0	0	0	0	0	0	0	1	0	0	0
101	10146	3	0	2	5	1	0	0	0	0	0	0	0	0	0	0	0	0	1	0	0	0
102	10147	3	1	4	2	2	1	0	1	0	0	0	1	0	0	0	1	0	2	0	1	0
103	10148	3	1	1	4	2	1	0	0	0	0	0	0	0	0	0	0	0	2	0	0	0
104	10149	3	0	2	3	3	1	0	1	0	0	1	0	0	0	1	1	0	2	0	1	0
105	101410	3	0	2	3	2	1	0	0	0	0	0	0	0	0	0	0	0	2	0	0	0
106	101411	3	1	1	5	1	0	0	0	0	0	0	0	0	0	0	0	0	1	0	0	0
107	101412	3	1	2	3	2	1	0	0	0	0	0	0	0	0	0	0	0	2	0	0	0
108	101413	3	1	4	2	2	1	0	1	0	0	0	1	0	1	1	1	1	2	0	1	0
109	101414	3	1	3	3	1	0	0	0	0	0	0	0	0	0	0	0	0	1	0	0	0
110	101415	3	1	1	4	1	0	0	0	0	0	0	0	0	0	0	0	0	1	0	0	0
111	101416	3	0	1	4	1	0	0	0	0	0	0	0	0	0	0	0	0	1	0	0	0
112	101417	3	1	2	3	2	1	0	1	0	0	0	1	0	1	0	1	1	2	0	1	0
113	101418	3	1	2	4	1	1	0	1	0	0	0	1	0	0	0	1	0	2	0	1	0
114	101419	3	1	2	3	2	1	1	1	0	0	1	0	0	0	0	1	0	2	0	1	0
115	101420	3	0	3	2	2	1	0	1	0	0	0	1	0	1	0	1	1	2	0	1	0
116	101421	3	1	2	4	2	0	0	0	0	0	0	0	0	0	0	0	0	1	0	0	0
117	101422	3	0	1	3	1	0	0	0	0	0	0	0	0	0	0	0	0	1	0	0	0
118	101423	3	1	2	4	2	1	0	1	0	0	0	0	1	0	1	0	2	0	1	0	
119	101424	3	0	4	1	2	1	1	1	1	1	0	1	0	1	0	1	1	3	1	1	1

120	101425	3	1	4	1	2	1	1	1	0	0	0	1	0	1	0	1	1	2	0	1	0
121	101426	3	0	3	1	1	1	1	1	1	1	0	0	1	0	1	1	0	3	1	1	1
122	101427	3	0	3	3	2	1	1	1	0	1	1	1	0	0	0	1	1	3	1	1	0
123	101428	3	0	1	4	2	1	0	0	0	0	0	0	0	0	0	0	0	2	0	0	0
124	101429	3	1	3	2	2	1	0	1	0	0	0	1	0	1	0	1	1	2	0	1	0
125	101430	3	0	2	3	2	1	0	1	0	0	0	0	0	1	0	1	0	2	0	1	0
126	101431	3	1	2	4	2	1	1	1	0	1	1	1	0	1	0	1	1	3	1	1	1
127	101432	3	1	1	4	2	1	1	1	0	0	0	0	1	0	0	1	0	2	0	1	0
128	101433	3	0	3	1	2	1	0	1	1	1	0	1	0	1	0	1	1	3	1	1	1
129	101434	3	1	3	2	1	1	1	1	0	0	1	0	0	1	1	1	1	3	1	1	0
130	101435	3	1	3	2	2	1	1	1	1	1	0	1	0	0	0	1	0	3	1	1	0
131	10151	3	0	4	2	2	1	1	1	1	0	0	0	0	1	0	1	0	2	0	1	0
132	10152	3	1	4	4	2	1	1	1	1	1	1	0	0	0	0	1	0	3	1	1	0
133	10153	3	0	3	2	2	1	1	1	1	1	0	1	1	0	0	1	1	3	1	1	1
134	10154	3	0	4	1	2	1	1	1	1	1	0	0	0	1	0	1	0	3	1	1	0
135	10155	3	1	3	3	2	1	1	1	1	1	0	0	0	1	1	1	0	3	1	1	1
136	10156	3	1	3	3	2	1	1	1	0	1	0	0	0	1	0	1	0	2	0	1	0
137	10157	3	0	4	1	2	1	1	1	1	1	0	1	0	1	0	1	1	3	1	1	1
138	10158	3	0	4	2	2	1	1	1	1	1	0	1	0	1	0	1	1	3	1	1	1
139	10159	3	1	4	5	3	1	1	1	1	1	0	1	0	1	0	1	1	3	1	1	1
140	101510	3	1	4	1	2	1	1	1	1	1	0	1	0	1	0	1	1	3	1	1	1
141	101511	3	0	4	4	3	1	1	1	1	1	0	0	1	1	0	1	1	3	1	1	1
142	101512	3	0	5	2	1	1	1	1	1	1	0	0	0	1	0	1	0	3	1	1	0
143	101513	3	1	4	3	2	1	1	1	1	1	0	0	1	0	0	1	0	3	1	1	0
144	101514	3	1	3	5	2	1	1	1	0	1	0	1	0	1	1	1	1	3	1	1	1
145	101515	3	0	4	1	2	1	1	1	0	1	0	1	0	0	0	1	0	2	0	1	0
146	101516	3	1	2	2	2	1	1	1	1	0	0	0	1	0	0	1	0	2	0	1	0
147	101517	3	0	3	2	2	1	1	1	1	1	1	0	0	0	0	1	0	3	1	1	0
148	101518	3	1	4	3	2	1	1	1	1	1	0	1	0	0	0	1	0	3	1	1	0
149	101519	3	0	4	2	2	1	1	1	0	1	0	0	0	1	0	1	0	2	0	1	0
150	101520	3	1	4	4	3	1	1	1	1	1	0	0	1	0	0	1	0	3	1	1	0
151	101521	3	0	2	5	2	1	1	1	1	0	0	0	0	1	0	1	0	2	0	1	0
152	101522	3	1	3	5	2	1	1	1	1	1	0	1	0	0	0	1	0	3	1	1	0
153	101523	3	1	4	4	2	1	1	1	1	1	0	0	0	0	0	0	0	2	0	1	0
154	101524	3	0	4	3	3	1	0	1	1	1	0	0	0	1	0	1	0	2	0	1	0
155	101525	3	1	4	2	2	1	1	1	1	1	0	0	0	1	0	1	0	3	1	1	0
156	101526	3	1	4	2	2	1	0	1	1	0	0	0	1	1	0	1	1	2	0	1	0
157	101527	3	1	4	3	2	1	0	1	1	1	0	1	0	1	0	1	1	3	1	1	1
158	101528	3	1	2	4	2	1	1	1	1	0	0	0	1	0	0	1	0	2	0	1	0
159	101529	3	0	3	3	2	1	1	1	1	1	1	0	0	0	0	1	0	3	1	1	0
160	101530	3	0	4	3	2	1	1	1	1	1	0	1	0	1	0	1	1	3	1	1	1
161	10161	3	0	4	1	2	1	1	1	0	0	0	0	0	1	0	1	0	2	0	1	0
162	10162	3	0	2	1	2	1	0	1	1	0	0	0	0	1	0	1	0	2	0	1	0
163	10163	3	0	4	2	3	1	1	1	1	1	0	0	0	1	0	1	0	3	1	1	0
164	10164	3	1	5	4	3	1	1	1	1	0	0	0	0	0	1	0	0	2	0	1	0
165	10165	3	1	2	2	2	1	0	1	1	1	0	0	0	1	0	1	0	2	0	1	0
166	10166	3	1	2	5	2	1	1	1	1	0	0	0	0	0	0	0	0	2	0	1	0
167	10167	3	1	2	4	2	1	0	1	1	1	0	0	1	0	0	1	0	2	0	1	0
168	10168	3	1	2	1	2	1	0	1	0	0	0	0	1	0	0	1	0	2	0	1	0
169	10169	3	0	2	3	2	1	0	1	1	1	0	0	0	1	0	1	0	2	0	1	0
170	101610	3	0	1	3	2	1	1	1	0	1	0	0	0	0	0	0	0	2	0	1	0
171	101611	3	0	2	1	2	1	1	1	1	1	0	0	0	1	0	1	0	3	1	1	0
172	101612	3	0	2	4	2	1	0	1	0	1	0	0	0	0	0	0	0	2	0	1	0
173	101613	3	1	2	3	2	1	1	1	1	1	1	0	0	0	0	1	0	3	1	1	0
174	101614	3	0	2	3	3	1	1	1	1	1	0	0	0	1	0	1	0	3	1	1	0
175	101615	3	0	2	4	2	1	1	1	1	0	0	0	0	0	0	0	0	2	0	1	0
176	101616	3	0	4	1	2	1	1	1	0	0	0	0	0	1	0	1	0	2	0	1	0
177	101617	3	0	1	3	2	1	1	1	1	1	0	0	0	1	0	1	0	3	1	1	0
178	101618	3	1	2	5	1	1	1	1	0	0	0	0	0	1	0	1	0	2	0	1	0
179	101619	3	1	2	4	2	1	1	1	1	1	0	0	0	1	0	1	0	3	1	1	0

180	101620	3	0	3	1	2	1	0	1	0	1	0	0	0	1	0	1	0	2	0	1	0
181	101621	3	1	2	4	2	1	1	1	0	1	0	0	1	1	0	1	1	3	1	1	0
182	101622	3	0	3	2	2	1	1	1	1	1	1	0	0	0	0	1	0	3	1	1	0
183	101623	3	1	2	1	2	1	0	1	0	0	0	0	0	1	0	1	0	2	0	1	0
184	101624	3	0	2	5	1	1	1	1	0	0	0	0	1	0	0	1	0	2	0	1	0
185	101625	3	1	3	2	2	1	1	1	0	0	0	0	0	1	0	1	0	2	0	1	0
186	101626	3	1	2	4	2	1	1	1	0	1	0	0	0	1	0	1	0	2	0	1	0
187	101627	3	1	2	3	2	1	1	1	1	1	0	0	0	1	0	1	0	3	1	1	0
188	101628	3	1	2	2	2	1	1	1	0	0	0	0	1	0	0	1	0	2	0	1	0
189	101629	3	1	3	3	2	1	1	1	1	1	0	0	0	1	0	1	0	3	1	1	0
190	101630	3	0	2	4	2	1	1	1	1	1	0	0	0	1	0	1	0	3	1	1	0
191	10271	3	0	1	3	2	1	0	1	0	0	0	1	0	0	0	1	0	2	0	1	0
192	10272	3	0	4	1	2	1	1	1	0	1	0	1	0	0	0	1	0	2	0	1	0
193	10273	3	0	2	2	2	1	1	1	0	0	0	1	0	0	0	1	0	2	0	1	0
194	10274	3	1	2	2	2	1	0	1	0	0	0	0	0	1	0	1	0	2	0	1	0
195	10275	3	0	2	4	1	1	0	1	0	0	0	1	0	1	1	1	1	2	0	1	0
196	10276	3	1	1	2	2	1	0	0	0	0	0	0	0	0	0	0	0	2	0	0	0
197	10277	3	0	3	1	2	1	0	1	1	1	0	1	0	1	0	1	1	3	1	1	1
198	10278	3	0	1	5	1	0	0	0	0	0	0	0	0	0	0	0	0	1	0	0	0
199	10279	3	1	2	4	2	1	1	1	1	1	0	1	1	1	1	1	1	4	1	1	1
200	102710	3	1	4	3	2	1	0	1	0	0	0	1	0	1	0	1	1	2	0	1	0
201	102711	3	1	1	4	2	1	0	0	0	0	0	0	0	0	0	0	0	2	0	0	0
202	102712	3	0	4	2	2	1	1	1	1	1	0	1	1	0	1	1	1	4	1	1	1
203	102713	3	0	2	4	2	0	0	0	0	0	0	0	0	0	0	0	0	1	0	0	0
204	102714	3	0	3	3	1	1	0	0	0	0	0	0	0	0	0	0	0	2	0	0	0
205	102715	3	1	1	5	1	0	0	0	0	0	0	0	0	0	0	0	0	1	0	0	0
206	102716	3	1	2	4	1	1	0	1	0	0	0	1	0	0	0	1	0	2	0	1	0
207	102717	3	0	2	4	1	0	0	0	0	0	0	0	0	0	0	0	0	1	0	0	0
208	102718	3	1	2	3	1	1	0	1	0	1	0	1	0	1	1	1	1	3	1	1	1
209	102719	3	0	1	4	1	1	1	1	1	0	0	0	0	1	0	1	0	2	0	1	0
210	102720	3	1	3	3	2	1	0	1	0	1	0	1	0	1	0	1	1	2	0	1	0
211	102721	3	1	2	4	2	1	1	1	0	1	0	1	1	1	1	1	1	4	1	1	1
212	102722	3	0	1	4	1	0	0	0	0	0	0	0	0	0	0	0	0	1	0	0	0
213	102723	3	0	2	2	1	1	0	0	0	0	0	0	0	0	0	0	0	2	0	0	0
214	102724	3	1	1	3	2	1	1	1	0	0	0	1	1	1	0	1	1	3	1	1	0
215	102725	3	0	2	2	1	1	0	1	0	0	0	1	0	1	0	1	1	2	0	1	0
216	102726	3	1	2	3	2	1	0	0	0	0	0	0	0	0	0	0	0	2	0	0	0
217	102727	3	1	2	2	2	1	0	1	0	0	0	1	0	1	1	1	1	2	0	1	0
218	102728	3	1	4	2	2	1	1	1	1	1	0	1	0	1	1	1	1	4	1	1	1
219	102729	3	1	3	2	2	1	0	1	0	1	0	1	1	1	0	1	1	3	1	1	1
220	102730	3	1	2	3	2	1	0	1	0	1	1	0	1	0	0	1	1	2	0	1	0
221	10281	3	1	2	3	2	1	1	1	0	0	0	1	0	0	0	1	0	2	0	1	0
222	10282	3	1	2	4	1	0	0	0	0	0	0	0	0	0	0	0	0	1	0	0	0
223	10283	3	0	1	4	1	1	1	1	0	0	0	0	0	0	0	0	0	2	0	1	0
224	10284	3	1	1	3	2	1	0	1	0	0	0	0	0	0	0	0	0	2	0	1	0
225	10285	3	1	2	4	2	1	0	1	0	1	0	1	0	0	1	1	0	2	0	1	0
226	10286	3	0	2	3	1	1	0	0	0	0	0	0	0	0	0	0	0	2	0	0	0
227	10287	3	0	1	5	1	0	0	0	0	0	0	0	0	0	0	0	0	1	0	0	0
228	10288	3	1	1	5	1	0	0	0	0	0	0	0	0	0	0	0	0	1	0	0	0
229	10289	3	1	3	1	1	1	0	1	0	1	0	1	0	0	0	1	0	2	0	1	0
230	102810	3	0	2	3	1	1	0	1	0	0	0	1	0	0	0	1	0	2	0	1	0
231	102811	3	1	3	3	2	1	0	1	1	1	0	1	0	0	1	1	0	3	1	1	1
232	102812	3	1	3	3	2	1	1	1	0	1	1	0	0	0	1	1	0	3	1	1	0
233	102813	3	1	2	4	1	1	0	1	0	0	0	0	1	0	0	1	0	2	0	1	0
234	102814	3	0	2	3	1	0	0	0	0	0	0	0	0	0	0	0	0	1	0	0	0
235	102815	3	1	1	5	1	0	0	0	0	0	0	0	0	0	0	0	0	1	0	0	0
236	102816	3	1	2	3	1	1	0	1	0	1	0	1	0	0	1	1	0	2	0	1	0
237	102817	3	0	2	4	1	0	0	0	0	0	0	0	0	0	1	0	0	2	0	0	0
238	102818	3	0	2	4	1	1	0	1	0	0	0	1	0	0	0	1	0	2	0	1	0
239	102819	3	1	2	3	1	1	0	1	0	0	0	1	0	1	0	1	1	2	0	1	0

240	102820	3	0	3	2	1	1	0	1	0	0	0	0	0	1	0	1	0	2	0	1	0
241	102821	3	1	3	2	3	1	1	1	1	1	1	1	0	1	0	1	1	4	1	1	1
242	102822	3	0	2	3	2	1	0	1	0	1	1	1	0	1	0	1	1	3	1	1	1
243	102823	3	0	2	3	3	1	1	1	1	1	0	1	0	0	1	1	0	3	1	1	1
244	102824	3	1	1	4	2	0	0	0	0	0	0	0	0	0	0	0	0	1	0	0	0
245	102825	3	1	2	4	2	1	0	1	0	0	0	0	0	0	1	0	0	2	0	1	0
246	102826	3	0	2	4	2	1	0	1	0	1	0	1	0	0	1	1	0	2	0	1	0
247	102827	3	0	2	5	3	1	1	1	1	1	1	1	1	0	1	1	1	4	1	1	1
248	102828	3	0	2	2	3	1	1	1	1	1	1	1	0	0	1	1	1	4	1	1	1
249	102829	3	1	1	5	3	1	0	1	0	1	1	1	1	0	1	1	1	3	1	1	1
250	102830	3	0	3	1	2	1	1	1	1	1	0	1	1	0	1	1	1	4	1	1	1
251	10291	3	1	2	5	2	1	0	1	0	1	0	1	1	0	1	1	1	3	1	1	1
252	10292	3	0	2	4	2	0	0	0	0	0	0	0	0	0	0	0	0	1	0	0	0
253	10293	3	0	1	5	2	0	0	0	0	0	0	0	0	0	0	0	0	1	0	0	0
254	10294	3	1	3	1	2	1	0	1	1	1	0	1	0	0	1	1	0	3	1	1	1
255	10295	3	1	3	2	2	1	0	1	0	0	0	0	0	1	1	1	0	2	0	1	0
256	10296	3	1	2	5	3	1	0	1	1	1	0	1	0	0	0	1	0	2	0	1	0
257	10297	3	1	2	3	2	0	0	0	0	0	0	0	0	0	0	0	0	1	0	0	0
258	10298	3	0	4	1	3	1	0	1	1	1	0	1	0	0	0	1	0	2	0	1	0
259	10299	3	1	2	4	2	1	0	1	0	1	0	0	0	0	0	0	0	2	0	1	0
260	102910	3	0	2	3	2	1	0	1	0	0	0	0	0	1	1	1	0	2	0	1	0
261	102911	3	1	3	3	2	1	0	1	0	1	0	0	0	1	0	1	0	2	0	1	0
262	102912	3	0	3	1	2	1	1	1	0	0	0	1	0	0	0	1	0	2	0	1	0
263	102913	3	1	2	3	2	1	1	1	1	1	1	1	1	1	0	1	1	4	1	1	1
264	102914	3	0	2	4	2	1	0	0	0	0	0	0	0	0	1	0	0	2	0	0	0
265	102915	3	0	1	4	2	1	0	0	0	0	0	0	0	0	0	0	0	2	0	0	0
266	102916	3	1	2	4	2	0	0	0	0	0	0	0	0	0	0	0	0	1	0	0	0
267	102917	3	1	2	3	2	1	0	1	0	0	0	0	0	0	1	0	0	2	0	1	0
268	102918	3	0	2	4	2	0	0	0	0	0	0	0	0	0	1	0	0	2	0	0	0
269	102919	3	0	2	4	2	0	0	0	0	0	0	0	0	0	0	0	0	1	0	0	0
270	102920	3	0	3	1	2	1	1	1	0	0	0	1	0	0	0	1	0	2	0	1	0
271	102921	3	0	2	1	2	1	0	1	1	1	0	1	0	0	0	1	0	2	0	1	0
272	102922	3	1	2	3	2	1	0	0	0	0	0	0	0	0	0	0	0	2	0	0	0
273	102923	3	1	2	3	2	1	0	1	0	0	0	0	0	0	1	0	0	2	0	1	0
274	102924	3	1	3	2	2	0	0	0	0	0	0	0	0	0	1	0	0	2	0	0	0
275	102925	3	0	4	2	3	1	1	1	1	1	0	1	1	1	0	1	1	4	1	1	1
276	102926	3	1	2	3	2	1	0	0	0	0	0	0	0	0	0	0	0	2	0	0	0
277	102927	3	1	2	3	2	0	0	0	0	0	0	0	0	0	1	0	0	2	0	0	0
278	102928	3	0	3	4	2	1	0	0	0	0	0	0	0	0	0	0	0	2	0	0	0
279	102929	3	0	2	4	2	1	0	0	1	0	0	0	0	0	0	0	0	2	0	0	0
280	102930	3	1	2	4	2	1	0	1	0	0	0	1	0	0	0	1	0	2	0	1	0
281	102101	3	0	4	1	2	1	1	1	1	1	1	1	1	0	1	1	1	4	1	1	1
282	102102	3	0	3	2	2	1	1	1	1	1	1	1	0	0	0	1	1	3	1	1	1
283	102103	3	0	2	2	2	1	1	1	1	1	0	1	0	0	0	1	0	3	1	1	0
284	102104	3	0	1	5	2	1	0	1	1	1	1	0	1	0	0	1	1	3	1	1	1
285	102105	3	1	3	5	3	1	1	1	1	1	0	1	0	0	1	1	0	3	1	1	1
286	102106	3	1	1	4	2	1	0	1	1	1	0	1	0	0	0	1	0	2	0	1	0
287	102107	3	0	4	1	2	1	1	1	1	1	0	1	0	0	0	1	0	3	1	1	0
288	102108	3	0	2	2	2	1	1	1	1	1	0	1	0	0	0	1	0	3	1	1	0
289	102109	3	1	2	3	3	1	1	1	1	1	0	1	0	0	0	1	0	3	1	1	0
290	1021010	3	0	2	3	2	1	1	1	1	1	1	1	0	0	0	1	1	3	1	1	1
291	1021011	3	1	1	4	2	1	0	1	1	1	0	1	0	0	0	1	0	2	0	1	0
292	1021012	3	0	2	1	3	1	1	1	1	1	0	1	0	0	0	1	0	3	1	1	0
293	1021013	3	0	1	5	2	1	0	1	0	1	0	0	0	0	0	0	0	2	0	1	0
294	1021014	3	0	2	2	2	1	0	1	1	1	0	1	0	0	0	1	0	2	0	1	0
295	1021015	3	1	1	5	2	1	1	1	1	1	0	1	0	0	1	1	0	3	1	1	1
296	1021016	3	1	2	4	2	1	0	1	1	1	1	0	0	0	0	1	0	2	0	1	0
297	1021017	3	1	3	2	2	1	1	1	1	1	0	1	0	0	0	1	0	3	1	1	0
298	1021018	3	1	1	2	2	1	0	1	1	1	1	1	0	0	0	1	1	3	1	1	1
299	1021019	3	1	1	4	2	1	0	1	1	1	0	1	0	0	0	1	0	2	0	1	0

300	1021020	3	1	2	5	2	1	0	1	1	1	0	1	0	0	0	1	0	2	0	1	0
301	1021021	3	1	1	4	2	1	0	1	1	1	0	0	1	0	0	1	0	2	0	1	0
302	1021022	3	1	1	3	2	0	0	0	0	0	0	0	0	0	0	0	0	1	0	0	0
303	1021023	3	1	2	4	3	1	1	1	1	1	0	0	1	0	0	1	0	3	1	1	0
304	1021024	3	1	1	3	2	1	1	1	1	1	0	1	0	0	0	1	0	3	1	1	0
305	1021025	3	1	2	4	2	1	1	1	1	1	0	1	0	0	0	1	0	3	1	1	0
306	1021026	3	1	2	4	2	1	0	1	1	1	1	0	0	0	0	1	0	2	0	1	0
307	1021027	3	0	1	5	1	1	0	0	0	0	0	0	0	0	0	0	0	2	0	0	0
308	1021028	3	0	1	5	2	1	1	0	1	1	0	0	0	1	1	1	0	3	1	1	1
309	1021029	3	0	1	5	2	1	0	1	1	1	0	1	0	0	1	1	0	3	1	1	1
310	1021030	3	0	2	5	3	1	0	1	1	1	0	1	0	0	1	1	0	3	1	1	1
311	1021031	3	0	3	2	2	1	1	1	0	1	0	1	0	1	0	1	1	3	1	1	0
312	1021032	3	0	4	2	3	1	1	1	1	1	0	1	0	0	0	1	0	3	1	1	0
313	1021033	3	0	3	2	2	1	1	1	1	1	0	1	0	0	1	1	0	3	1	1	1
314	102111	3	0	2	3	1	0	0	0	0	0	0	0	0	0	0	0	0	1	0	0	0
315	102112	3	1	2	3	2	1	0	1	0	1	0	1	0	0	0	1	0	2	0	1	0
316	102113	3	0	1	4	2	1	0	1	1	1	0	1	0	0	0	1	0	2	0	1	0
317	102114	3	0	3	3	2	1	0	1	0	1	0	1	0	0	0	1	0	2	0	1	0
318	102115	3	1	2	2	2	1	0	1	1	0	0	1	0	0	1	1	0	2	0	1	0
319	102116	3	0	2	2	2	1	1	1	1	0	0	1	0	0	0	1	0	2	0	1	0
320	102117	3	1	5	2	3	1	1	1	1	1	0	1	1	1	1	1	1	4	1	1	1
321	102118	3	1	3	1	2	1	1	1	1	1	0	1	1	1	0	1	1	4	1	1	1
322	102119	3	1	4	5	2	1	1	1	1	1	0	1	1	1	1	1	1	4	1	1	1
323	1021110	3	0	1	4	2	0	0	0	0	0	0	0	0	0	0	0	0	1	0	0	0
324	1021111	3	1	4	2	2	1	1	1	1	0	0	1	0	0	0	1	0	2	0	1	0
325	1021112	3	1	3	2	2	1	1	1	0	1	0	1	0	1	1	1	1	3	1	1	1
326	1021113	3	1	1	1	2	1	0	0	0	0	0	0	0	0	0	0	0	2	0	0	0
327	1021114	3	0	1	3	1	1	0	0	0	0	0	0	0	0	0	0	0	2	0	0	0
328	1021115	3	0	2	3	2	1	1	1	0	1	0	1	0	0	0	1	0	2	0	1	0
329	1021116	3	0	2	3	2	1	0	1	1	1	0	1	0	0	0	1	0	2	0	1	0
330	1021117	3	1	2	5	2	1	1	1	1	1	0	1	0	0	1	1	0	3	1	1	1
331	1021118	3	0	2	4	2	1	1	1	1	1	0	1	0	1	1	1	1	4	1	1	1
332	1021119	3	0	2	4	2	1	0	1	1	1	0	1	0	0	1	1	0	3	1	1	1
333	1021120	3	1	1	3	2	1	0	1	1	1	0	1	0	1	0	1	1	3	1	1	1
334	1021121	3	1	1	5	1	0	0	0	0	0	0	0	0	0	0	0	0	1	0	0	0
335	1021122	3	1	1	5	3	1	1	1	1	1	0	1	0	0	0	1	0	3	1	1	0
336	1021123	3	0	2	5	1	1	0	1	1	1	0	0	0	0	0	0	0	2	0	1	0
337	1021124	3	1	1	4	2	1	1	1	1	1	0	1	0	0	0	1	0	3	1	1	0
338	1021125	3	1	3	2	3	1	1	1	1	1	0	1	0	0	0	1	0	3	1	1	0
339	1021126	3	0	2	4	3	1	1	1	1	1	0	1	0	0	0	1	0	3	1	1	0
340	1021127	3	1	1	3	2	1	1	1	1	1	0	0	1	1	1	1	1	4	1	1	1
341	1021128	3	1	4	3	3	1	1	1	1	1	0	0	1	1	1	1	1	4	1	1	1
342	1021129	3	1	4	3	3	1	1	1	1	1	0	0	1	1	0	1	1	3	1	1	1
343	1021130	3	1	4	3	3	1	1	1	1	1	0	1	1	1	1	1	1	4	1	1	1
344	1021131	3	0	4	2	2	1	1	1	1	1	0	1	1	1	1	1	1	4	1	1	1
345	1021132	3	0	4	3	3	1	1	1	1	1	0	1	1	1	0	1	1	4	1	1	1
346	1021133	3	0	2	4	3	1	1	1	1	1	0	1	0	0	1	1	0	3	1	1	1
347	1021134	3	1	4	4	3	1	1	1	1	1	0	1	1	1	1	1	1	4	1	1	1
348	1021135	3	1	5	2	3	1	1	1	1	1	0	1	1	1	1	1	1	4	1	1	1
349	1021136	3	0	4	2	2	1	1	1	1	1	0	1	1	1	1	1	1	4	1	1	1
350	1021137	3	0	2	5	2	1	1	1	1	1	0	1	0	0	1	1	0	3	1	1	1
351	1021138	3	1	5	2	3	1	1	1	1	1	0	1	1	1	1	1	1	4	1	1	1
352	1021139	3	0	2	4	2	1	0	1	1	1	0	0	1	0	1	1	0	3	1	1	1
353	102121	3	0	1	5	2	0	0	0	0	0	0	0	0	0	0	0	0	1	0	0	0
354	102122	3	0	2	4	3	1	0	1	1	1	0	1	0	0	1	1	0	3	1	1	1
355	102123	3	1	5	5	3	1	0	1	1	1	0	1	1	0	1	1	1	3	1	1	1
356	102124	3	1	1	4	2	0	0	0	0	0	0	0	0	0	0	0	0	1	0	0	0
357	102125	3	0	3	3	2	1	1	1	0	1	0	1	0	0	0	1	0	2	0	1	0
358	102126	3	1	2	3	2	1	1	1	0	0	0	0	0	0	0	0	0	2	0	1	0
359	102127	3	0	1	3	2	1	0	1	1	1	0	1	0	1	0	1	1	3	1	1	1

360	102128	3	1	3	3	3	1	0	1	1	1	0	1	0	0	0	1	0	2	0	1	0
361	102129	3	0	4	1	3	1	0	1	1	1	0	1	1	1	0	1	1	3	1	1	1
362	1021210	3	1	2	3	3	1	1	1	1	1	0	1	0	0	1	1	0	3	1	1	1
363	1021211	3	0	5	1	3	1	1	1	1	1	0	1	1	1	1	1	1	4	1	1	1
364	1021212	3	1	2	2	3	1	1	1	1	1	0	1	0	0	0	1	0	3	1	1	0
365	1021213	3	0	2	5	2	1	1	1	1	1	0	1	1	1	1	1	1	4	1	1	1
366	1021214	3	0	2	4	2	1	1	1	1	1	0	1	1	1	0	1	1	4	1	1	1
367	1021215	3	0	2	4	2	1	1	1	1	1	0	1	0	0	0	1	0	3	1	1	0
368	1021216	3	1	1	5	2	1	1	1	1	1	0	1	0	0	0	1	0	3	1	1	0
369	1021217	3	0	2	4	2	1	1	1	1	1	0	1	0	0	0	1	0	3	1	1	0
370	1021218	3	1	3	1	3	1	0	1	1	1	0	1	0	1	0	1	1	3	1	1	1
371	1021219	3	1	3	3	2	1	0	1	1	1	0	1	1	1	1	1	1	4	1	1	1
372	1021220	3	1	2	4	2	1	1	1	1	1	0	1	1	1	0	1	1	4	1	1	1
373	1021221	3	1	2	5	3	1	0	1	1	1	0	1	1	1	1	1	1	4	1	1	1
374	1021222	3	0	1	4	2	1	1	1	1	1	0	1	0	0	1	1	0	3	1	1	1
375	1021223	3	1	5	2	3	1	1	1	1	1	0	1	1	1	0	1	1	4	1	1	1
376	1021224	3	0	5	4	3	1	1	1	1	1	0	1	1	1	0	1	1	4	1	1	1
377	1021225	3	0	2	5	2	1	1	1	1	1	0	1	1	1	0	1	1	4	1	1	1
378	1021226	3	0	1	4	2	1	0	0	1	1	0	0	0	0	0	0	0	2	0	0	0
379	1021227	3	1	1	2	2	1	1	1	1	1	0	1	1	1	1	1	1	4	1	1	1
380	1021228	3	1	1	5	2	1	0	1	1	1	0	1	0	0	0	1	0	2	0	1	0
381	1021229	3	0	2	2	2	1	1	1	1	1	0	1	1	0	0	1	1	3	1	1	1
382	1021230	3	1	2	2	2	1	0	1	1	1	0	1	0	0	0	1	0	2	0	1	0
383	1021231	3	1	1	3	2	1	1	1	1	1	0	1	0	1	0	1	1	3	1	1	1
384	1021232	3	1	1	4	2	1	1	1	1	1	0	1	1	1	0	1	1	4	1	1	1
385	1021233	3	1	5	1	3	1	1	1	1	1	0	1	1	1	0	1	1	4	1	1	1
386	1021234	3	0	1	5	2	1	1	1	1	1	0	1	0	0	1	1	0	3	1	1	1
387	1021235	3	0	5	2	2	1	1	1	1	1	0	1	1	1	1	1	1	4	1	1	1
388	103141	3	1	3	4	2	0	0	0	0	0	0	0	0	0	1	0	0	2	0	0	0
389	103142	3	1	3	2	2	0	0	0	0	0	0	0	0	0	0	0	0	1	0	0	0
390	103143	3	1	3	5	2	0	0	0	0	0	0	0	0	0	0	0	0	1	0	0	0
391	103144	3	1	3	3	2	1	0	1	0	1	0	1	0	0	1	1	0	2	0	1	0
392	103145	3	1	3	3	2	0	0	0	0	0	0	0	0	0	1	0	0	2	0	0	0
393	103146	3	1	1	5	2	0	0	0	0	0	0	0	0	0	1	0	0	2	0	0	0
394	103147	3	1	1	5	1	0	0	0	0	0	0	0	0	0	1	0	0	2	0	0	0
395	103148	3	1	3	4	2	0	0	0	0	0	0	0	0	0	1	0	0	2	0	0	0
396	103149	3	1	2	3	2	0	0	0	0	0	0	0	0	0	1	0	0	2	0	0	0
397	1031410	3	1	4	4	2	1	0	1	0	1	0	1	0	0	1	1	0	2	0	1	0
398	1031411	3	0	2	5	2	0	0	0	0	0	0	0	0	0	1	0	0	2	0	0	0
399	1031412	3	0	2	3	1	0	0	0	0	0	0	0	0	0	1	0	0	2	0	0	0
400	1031413	3	0	1	4	2	0	0	0	0	0	0	0	0	0	1	0	0	2	0	0	0
401	1031414	3	0	2	3	2	0	0	0	0	0	0	0	0	0	0	0	0	1	0	0	0
402	1031415	3	0	1	3	2	1	0	1	0	1	0	1	0	0	1	1	0	2	0	1	0
403	1031416	3	0	2	3	2	0	0	0	0	0	0	0	0	0	1	0	0	2	0	0	0
404	1031417	3	0	2	5	1	0	0	0	0	0	0	0	0	0	1	0	0	2	0	0	0
405	1031418	3	0	2	5	2	0	0	0	0	0	0	0	0	0	1	0	0	2	0	0	0
406	1031419	3	0	2	4	1	0	0	0	0	0	0	0	0	0	1	0	0	2	0	0	0
407	1031420	3	0	2	5	2	0	0	0	0	0	0	0	0	0	1	0	0	2	0	0	0
408	1031421	3	0	2	4	1	0	0	0	0	0	0	0	0	0	1	0	0	2	0	0	0
409	1031422	3	0	2	4	1	0	0	0	0	0	0	0	0	0	1	0	0	2	0	0	0
410	1031423	3	0	2	1	1	1	0	1	0	1	0	1	0	0	1	1	0	2	0	1	0
411	1031424	3	0	1	4	2	0	0	0	0	0	0	0	0	0	1	0	0	2	0	0	0
412	1031425	3	1	1	4	1	0	0	0	0	0	0	0	0	0	1	0	0	2	0	0	0
413	1031426	3	0	2	2	1	0	0	0	0	0	0	0	0	0	1	0	0	2	0	0	0
414	1031427	3	0	2	3	1	1	0	1	0	1	0	1	0	0	1	1	0	2	0	1	0
415	1031428	3	0	4	1	1	0	0	0	0	0	0	0	0	0	1	0	0	2	0	0	0
416	1031429	3	0	3	4	2	1	0	1	0	1	0	1	0	0	0	1	0	2	0	1	0
417	1031430	3	0	2	3	1	0	0	0	0	0	0	0	0	0	1	0	0	2	0	0	0
418	103131	3	0	2	3	2	1	0	1	0	1	0	1	0	0	1	1	0	2	0	1	0
419	103132	3	0	5	2	2	1	0	1	0	1	0	1	0	0	1	1	0	2	0	1	0

420	103133	3	0	2	2	2	1	0	1	0	0	0	0	0	0	0	0	0	2	0	1	0
421	103134	3	0	3	2	3	1	0	1	0	1	0	1	0	0	1	1	0	2	0	1	0
422	103135	3	0	5	2	3	1	0	1	0	1	0	1	0	0	1	1	0	2	0	1	0
423	103136	3	1	2	4	1	1	0	0	0	0	0	0	0	0	0	0	0	2	0	0	0
424	103137	3	0	2	3	1	1	0	1	0	1	0	1	0	0	0	1	0	2	0	1	0
425	103138	3	0	2	2	1	0	0	0	0	0	0	0	0	0	1	0	0	2	0	0	0
426	103139	3	0	4	3	1	1	0	1	0	1	0	1	0	0	1	1	0	2	0	1	0
427	1031310	3	1	2	5	1	1	0	0	0	0	0	0	0	0	0	0	0	2	0	0	0
428	1031311	3	0	2	4	2	1	0	1	0	1	0	1	0	0	0	1	0	2	0	1	0
429	1031312	3	1	2	4	1	0	0	0	0	0	0	0	0	0	0	0	0	1	0	0	0
430	1031313	3	1	4	3	2	1	0	1	0	1	1	0	0	0	0	1	0	2	0	1	0
431	1031314	3	1	4	2	2	1	0	1	1	1	1	0	0	0	0	1	0	2	0	1	0
432	1031315	3	0	2	4	1	1	0	0	0	0	0	0	0	0	0	0	0	2	0	0	0
433	1031316	3	0	2	4	1	1	0	1	0	1	1	0	0	0	0	1	0	2	0	1	0
434	1031317	3	1	2	2	2	0	0	0	0	0	0	0	0	0	1	0	0	2	0	0	0
435	1031318	3	0	2	3	1	1	0	0	0	0	0	0	0	0	0	0	0	2	0	0	0
436	1031319	3	0	4	1	3	1	0	1	0	0	0	0	0	1	0	1	0	2	0	1	0
437	1031320	3	0	4	1	2	1	0	1	0	0	0	0	0	1	1	1	0	2	0	1	0
438	1031321	3	0	2	5	1	0	0	0	0	0	0	0	0	0	0	0	0	1	0	0	0
439	1031322	3	0	2	5	2	0	0	0	0	0	0	0	0	0	0	0	0	1	0	0	0
440	1031323	3	0	2	2	2	0	0	0	0	0	0	0	0	0	0	0	0	1	0	0	0
441	1031324	3	0	2	3	2	1	0	1	0	1	0	1	0	0	0	1	0	2	0	1	0
442	1031325	3	0	2	4	2	1	0	1	0	1	0	1	0	0	0	1	0	2	0	1	0
443	1031326	3	0	2	2	2	1	0	0	0	0	0	0	0	0	1	0	0	2	0	0	0
444	1031327	3	0	2	3	2	1	0	0	0	0	0	0	0	0	0	0	0	2	0	0	0
445	1031328	3	0	2	5	1	0	0	0	0	0	0	0	0	0	0	0	0	1	0	0	0
446	1031329	3	0	2	3	1	1	0	1	0	0	0	0	0	1	0	1	0	2	0	1	0
447	1031330	3	0	2	3	2	0	0	0	0	0	0	0	0	0	0	0	0	1	0	0	0
448	103151	3	0	1	4	1	0	0	0	0	0	0	0	0	0	0	0	0	1	0	0	0
449	103152	3	0	2	4	2	1	0	1	0	0	0	0	0	0	0	0	0	2	0	1	0
450	103153	3	0	2	4	2	0	0	0	0	0	0	0	0	0	1	0	0	2	0	0	0
451	103154	3	0	2	1	2	0	0	0	0	0	0	0	0	0	0	0	0	1	0	0	0
452	103155	3	0	2	2	2	1	0	1	0	0	0	1	0	0	0	1	0	2	0	1	0
453	103156	3	1	2	4	2	0	0	0	0	0	0	0	0	0	1	0	0	2	0	0	0
454	103157	3	0	4	1	1	0	0	0	0	0	0	0	0	0	1	0	0	2	0	0	0
455	103158	3	0	2	4	1	0	0	0	0	0	0	0	0	0	1	0	0	2	0	0	0
456	103159	3	0	2	5	2	0	0	0	0	0	0	0	0	0	0	0	0	1	0	0	0
457	1031510	3	0	2	3	2	0	0	0	0	0	0	0	0	0	1	0	0	2	0	0	0
458	1031511	3	0	3	2	2	0	0	0	0	0	0	0	0	0	1	0	0	2	0	0	0
459	1031512	3	0	2	2	1	0	0	0	0	0	0	0	0	0	0	0	0	1	0	0	0
460	1031513	3	0	4	1	1	0	0	0	0	0	0	0	0	0	1	0	0	2	0	0	0
461	1031514	3	0	3	1	1	0	0	0	0	0	0	0	0	0	0	0	0	1	0	0	0
462	1031515	3	0	4	1	1	0	0	0	0	0	0	0	0	0	1	0	0	2	0	0	0
463	1031516	3	0	3	2	1	1	0	1	0	1	0	1	0	0	0	1	0	2	0	1	0
464	1031517	3	0	2	4	1	0	0	0	0	0	0	0	0	0	0	0	0	1	0	0	0
465	1031518	3	0	3	4	2	1	0	1	0	1	0	1	0	0	1	1	0	2	0	1	0
466	1031519	3	0	4	1	1	1	0	1	0	1	0	1	0	0	1	1	0	2	0	1	0
467	1031520	3	1	2	4	2	0	0	0	0	0	0	0	0	0	0	0	0	1	0	0	0
468	1031521	3	0	1	4	1	0	0	0	0	0	0	0	0	0	0	0	0	1	0	0	0
469	1031522	3	1	2	5	2	0	0	0	0	0	0	0	0	0	0	0	0	1	0	0	0
470	1031523	3	1	2	5	3	1	0	1	0	1	0	1	0	0	1	1	0	2	0	1	0
471	1031524	3	0	1	5	2	0	0	0	0	0	0	0	0	0	0	0	0	1	0	0	0
472	1031525	3	1	1	4	2	0	0	0	0	0	0	0	0	0	0	0	0	1	0	0	0
473	1031526	3	0	1	5	1	0	0	0	0	0	0	0	0	0	0	0	0	1	0	0	0
474	1031527	3	0	2	4	2	0	0	0	0	0	0	0	0	0	0	0	0	1	0	0	0
475	1031528	3	0	3	1	2	1	0	1	0	1	0	1	0	0	1	1	0	2	0	1	0
476	1031529	3	0	2	3	2	1	0	1	0	1	0	1	0	0	0	1	0	2	0	1	0
477	1031530	3	0	5	2	3	1	0	1	0	1	0	1	0	0	1	1	0	2	0	1	0
478	103161	3	0	2	3	2	0	0	0	0	0	0	0	0	0	1	0	0	2	0	0	0
479	103162	3	0	3	3	2	0	0	0	0	0	0	0	0	0	1	0	0	2	0	0	0

480	103163	3	0	3	2	3	1	0	0	0	0	0	0	0	0	1	0	0	2	0	0	0
481	103164	3	0	3	2	2	1	0	0	0	0	0	0	0	0	1	0	0	2	0	0	0
482	103165	3	0	4	4	1	1	0	0	0	0	0	0	0	0	1	0	0	2	0	0	0
483	103166	3	0	2	4	1	1	0	0	0	0	0	0	0	0	1	0	0	2	0	0	0
484	103167	3	0	2	2	1	0	0	0	0	0	0	0	0	0	1	0	0	2	0	0	0
485	103168	3	0	2	5	2	1	0	1	0	1	0	1	0	0	1	1	0	2	0	1	0
486	103169	3	0	2	5	1	0	0	0	0	0	0	0	0	0	1	0	0	2	0	0	0
487	1031610	3	0	4	1	2	0	0	0	0	0	0	0	0	0	0	0	0	1	0	0	0
488	1031611	3	0	2	1	1	1	0	0	0	0	0	0	0	0	0	0	0	2	0	0	0
489	1031612	3	0	2	2	2	1	0	0	0	0	0	0	0	0	0	0	0	2	0	0	0
490	1031613	3	0	2	3	2	1	0	0	0	0	0	0	0	0	0	0	0	2	0	0	0
491	1031614	3	0	2	3	1	1	0	0	0	0	0	0	0	0	0	0	0	2	0	0	0
492	1031615	3	0	2	5	1	1	0	0	0	0	0	0	0	0	1	0	0	2	0	0	0
493	1031616	3	0	2	4	1	0	0	0	0	0	0	0	0	0	0	0	0	1	0	0	0
494	1031617	3	0	2	4	2	0	0	0	0	0	0	0	0	0	1	0	0	2	0	0	0
495	1031618	3	0	2	4	1	0	0	0	0	0	0	0	0	0	0	0	0	1	0	0	0
496	1031619	3	0	1	5	2	0	0	0	0	0	0	0	0	0	0	0	0	1	0	0	0
497	1031620	3	0	3	3	2	0	0	0	0	0	0	0	0	0	1	0	0	2	0	0	0
498	1031621	3	0	5	1	3	1	0	1	0	1	1	0	1	1	0	1	1	3	1	1	1
499	1031622	3	0	4	4	2	0	0	0	0	0	0	0	0	0	1	0	0	2	0	0	0
500	1031623	3	0	3	4	3	1	0	1	0	1	0	1	0	0	1	1	0	2	0	1	0
501	204171	1	0	4	1	2	1	1	1	0	1	0	1	1	0	1	1	1	3	1	1	1
502	204172	1	0	1	3	2	1	1	1	0	1	0	1	0	0	1	1	0	3	1	1	0
503	204173	1	0	2	1	2	1	1	1	0	1	0	0	0	1	0	1	0	2	0	1	0
504	204174	1	1	2	2	2	1	0	1	0	1	0	1	0	1	0	1	1	2	0	1	0
505	204175	1	0	4	1	2	1	1	1	1	1	0	1	0	0	0	1	0	3	1	1	0
506	204176	1	1	2	5	2	1	1	1	0	1	1	0	0	0	0	1	0	2	0	1	0
507	204177	1	1	2	4	1	1	0	1	1	1	0	1	0	0	1	1	0	3	1	1	1
508	204178	1	1	1	2	2	1	0	1	0	1	0	1	0	1	0	1	1	2	0	1	0
509	204179	1	0	2	1	2	1	0	1	1	1	0	1	0	0	0	1	0	2	0	1	0
510	2041710	1	0	5	1	2	1	0	1	1	1	0	0	1	1	1	1	1	3	1	1	1
511	2041711	1	1	2	4	2	1	0	1	0	1	0	1	1	0	0	1	1	2	0	1	0
512	2041712	1	1	2	4	2	1	1	1	0	1	0	1	0	1	0	1	1	3	1	1	0
513	2041713	1	0	2	4	2	1	1	1	1	1	0	1	1	0	1	1	1	4	1	1	1
514	2041714	1	1	2	5	1	1	1	1	1	0	0	1	0	1	0	1	1	3	1	1	0
515	2041715	1	1	2	5	2	1	0	1	0	0	0	1	0	0	0	1	0	2	0	1	0
516	2041716	1	1	2	5	2	1	0	1	0	0	0	1	0	0	0	1	0	2	0	1	0
517	2041717	1	1	3	4	3	1	1	1	1	1	0	1	1	1	1	1	1	4	1	1	1
518	2041718	1	0	1	4	2	1	0	1	0	1	0	1	1	0	0	1	1	2	0	1	0
519	2041719	1	0	2	3	2	1	0	1	1	0	0	1	1	0	0	1	1	2	0	1	0
520	2041720	1	1	2	5	2	1	1	1	0	1	0	0	1	1	0	1	1	3	1	1	0
521	2041721	1	1	1	4	2	1	0	1	0	1	0	1	0	0	0	1	0	2	0	1	0
522	2041722	1	1	1	2	2	1	0	1	0	1	0	1	1	0	0	1	1	2	0	1	0
523	2041723	1	0	3	2	2	1	0	1	0	1	0	0	0	0	1	0	0	2	0	1	0
524	2041724	1	0	1	5	2	1	1	1	1	1	0	1	0	0	0	1	0	3	1	1	0
525	2041725	1	1	1	5	2	1	1	1	0	0	0	1	0	0	1	1	0	2	0	1	0
526	2041726	1	1	2	2	2	1	0	1	0	0	0	1	0	0	0	1	0	2	0	1	0
527	2041727	1	1	1	5	2	1	0	1	0	1	0	1	1	0	0	1	1	2	0	1	0
528	2041728	1	1	2	3	3	1	1	1	1	1	0	1	0	1	1	1	1	4	1	1	1
529	2041729	1	1	2	2	2	1	0	1	0	1	0	1	0	0	0	1	0	2	0	1	0
530	2041730	1	0	2	4	1	1	0	1	1	1	0	1	0	0	0	1	0	2	0	1	0
531	2041731	1	1	2	1	2	1	1	1	0	1	0	1	0	0	1	1	0	3	1	1	0
532	2041732	1	1	5	2	2	1	1	1	1	0	0	1	0	0	0	1	0	2	0	1	0
533	2041733	1	1	3	2	2	1	1	1	1	0	0	1	0	1	0	1	1	3	1	1	0
534	2041734	1	1	2	4	1	1	1	1	1	0	0	1	0	1	0	1	1	3	1	1	0
535	2041735	1	0	2	3	2	1	0	1	1	1	0	0	0	1	0	1	0	2	0	1	0
536	204181	1	1	2	3	3	1	1	1	0	1	0	1	0	1	0	1	1	3	1	1	0
537	204182	1	1	2	4	1	1	1	1	0	0	0	0	0	0	0	0	0	2	0	1	0
538	204183	1	1	2	3	2	1	1	1	0	1	0	1	1	0	0	1	1	3	1	1	0
539	204184	1	1	2	4	2	1	0	1	0	1	0	1	1	0	0	1	1	2	0	1	0

540	204185	1	0	1	5	2	1	0	1	0	0	0	1	0	0	0	1	0	2	0	1	0
541	204186	1	0	1	5	2	1	0	1	0	1	0	1	0	0	0	1	0	2	0	1	0
542	204187	1	1	5	2	2	1	1	1	1	1	0	1	1	0	1	1	1	4	1	1	1
543	204188	1	1	4	1	2	1	1	1	0	0	0	1	0	0	0	1	0	2	0	1	0
544	204189	1	1	2	4	2	1	1	1	0	0	0	1	0	0	0	1	0	2	0	1	0
545	2041810	1	1	3	5	1	1	1	1	0	1	0	1	1	0	1	1	1	3	1	1	1
546	2041811	1	1	4	4	3	1	0	1	0	1	0	1	0	1	0	1	1	2	0	1	0
547	2041812	1	0	2	3	3	1	0	1	1	0	1	1	0	0	1	1	1	3	1	1	1
548	2041813	1	0	2	3	2	1	1	1	0	1	0	1	0	0	0	1	0	2	0	1	0
549	2041814	1	1	4	3	2	1	1	1	1	1	1	1	0	0	1	1	1	4	1	1	1
550	2041815	1	1	5	1	1	1	1	1	1	1	1	1	1	0	0	1	1	4	1	1	1
551	2041816	1	1	2	3	2	1	0	1	0	1	0	0	0	1	0	1	0	2	0	1	0
552	2041817	1	1	2	4	2	1	0	1	0	0	0	0	0	0	1	0	0	2	0	1	0
553	2041818	1	1	2	5	2	1	0	1	0	0	0	0	0	1	0	1	0	2	0	1	0
554	2041819	1	1	5	2	2	1	1	1	0	0	0	0	0	1	1	1	0	2	0	1	0
555	2041820	1	0	1	4	3	1	0	1	0	0	0	0	0	1	0	1	0	2	0	1	0
556	2041821	1	1	3	3	2	1	1	1	0	1	0	1	0	1	0	1	1	3	1	1	0
557	2041822	1	1	1	4	2	1	1	1	1	1	1	0	1	0	0	1	1	3	1	1	1
558	2041823	1	1	1	5	2	1	0	1	0	1	0	1	0	0	0	1	0	2	0	1	0
559	2041824	1	1	4	1	2	1	1	1	1	1	0	1	1	1	1	1	1	4	1	1	1
560	2041825	1	0	3	1	2	1	1	1	0	1	0	1	0	0	0	1	0	2	0	1	0
561	2041826	1	1	2	3	2	1	0	1	0	1	0	1	0	1	0	1	1	2	0	1	0
562	2041827	1	1	2	5	2	1	0	0	0	1	0	0	0	0	0	0	0	2	0	0	0
563	2041828	1	1	1	5	2	1	0	1	0	1	0	1	0	0	0	1	0	2	0	1	0
564	2041829	1	1	4	1	2	1	1	1	1	1	0	1	0	1	0	1	1	3	1	1	1
565	2041830	1	1	2	4	2	1	0	1	0	0	0	1	0	0	0	1	0	2	0	1	0
566	204191	1	1	4	2	2	1	1	1	0	1	0	0	1	0	1	1	0	3	1	1	0
567	204192	1	1	5	1	2	1	1	1	0	1	0	0	1	1	0	1	1	3	1	1	0
568	204193	1	0	2	2	1	1	1	1	0	0	0	0	1	1	0	1	1	2	0	1	0
569	204194	1	1	1	5	1	1	1	1	0	1	0	0	0	0	0	0	0	2	0	1	0
570	204195	1	0	2	3	2	1	1	1	0	1	0	0	1	0	1	1	0	3	1	1	0
571	204196	1	0	2	4	1	1	1	1	0	1	0	0	0	0	0	0	0	2	0	1	0
572	204197	1	0	1	5	1	1	0	1	0	1	0	0	1	0	0	1	0	2	0	1	0
573	204198	1	0	4	3	2	1	1	1	0	1	0	0	1	1	0	1	1	3	1	1	0
574	204199	1	0	1	5	1	0	0	0	0	0	0	0	0	0	0	0	0	1	0	0	0
575	2041910	1	0	2	3	1	1	1	1	0	1	0	0	0	1	0	1	0	2	0	1	0
576	2041911	1	0	2	3	2	1	1	1	0	0	0	0	0	0	0	0	0	2	0	1	0
577	2041912	1	1	3	2	2	1	1	1	0	1	0	0	1	1	0	1	1	3	1	1	0
578	2041913	1	1	1	5	1	1	1	1	0	1	0	0	1	0	0	1	0	2	0	1	0
579	2041914	1	1	1	5	1	1	1	1	1	1	0	0	1	0	0	1	0	3	1	1	0
580	2041915	1	0	2	3	1	1	1	1	0	1	0	0	1	0	0	1	0	2	0	1	0
581	2041916	1	1	2	2	2	1	1	1	0	1	0	0	1	1	0	1	1	3	1	1	0
582	2041917	1	1	2	2	1	1	1	1	0	1	0	0	0	1	0	1	0	2	0	1	0
583	2041918	1	1	2	3	2	1	0	1	0	1	0	0	1	0	0	1	0	2	0	1	0
584	2041919	1	1	2	3	1	1	1	1	0	1	0	0	1	1	0	1	1	3	1	1	0
585	2041920	1	0	1	5	1	1	1	1	0	1	0	0	0	0	0	0	0	2	0	1	0
586	2041921	1	0	2	3	2	1	1	1	0	1	0	0	1	1	1	1	1	3	1	1	1
587	2041922	1	1	2	4	1	1	0	1	0	1	0	0	0	1	0	1	0	2	0	1	0
588	2041923	1	1	1	5	1	1	0	1	0	1	0	0	0	1	0	1	0	2	0	1	0
589	2041924	1	1	2	2	2	1	1	1	0	1	0	0	0	1	0	1	0	2	0	1	0
590	2041925	1	1	2	2	1	1	0	1	0	1	0	0	0	0	1	0	0	2	0	1	0
591	2041926	1	0	3	2	1	1	1	1	0	1	0	0	1	1	1	1	1	3	1	1	1
592	2041927	1	1	1	4	2	1	1	1	0	1	0	1	1	0	0	1	1	3	1	1	0
593	2041928	1	0	1	4	1	1	0	1	0	1	0	0	0	1	1	1	0	2	0	1	0
594	2041929	1	0	4	1	1	1	1	1	1	1	0	0	1	1	0	1	1	3	1	1	1
595	2041930	1	1	2	2	1	1	1	1	0	1	0	0	1	1	0	1	1	3	1	1	0
596	204201	1	1	4	2	2	1	1	1	0	1	0	0	1	1	0	1	1	3	1	1	0
597	204202	1	0	4	1	2	1	1	1	0	1	0	0	1	0	0	1	0	2	0	1	0
598	204203	1	1	5	3	2	1	1	1	0	1	0	0	1	1	0	1	1	3	1	1	0
599	204204	1	1	2	2	1	1	1	1	0	1	0	0	1	1	0	1	1	3	1	1	0

600	204205	1	1	2	3	2	1	1	1	0	1	0	0	0	0	0	0	0	2	0	1	0
601	204206	1	1	1	5	1	0	0	0	0	0	0	0	0	0	0	0	0	1	0	0	0
602	204207	1	1	2	3	1	1	1	1	0	1	0	0	0	0	0	0	0	2	0	1	0
603	204208	1	1	3	1	2	1	1	1	0	1	0	0	0	0	0	0	0	2	0	1	0
604	204209	1	0	2	3	1	1	1	1	0	0	0	0	0	0	0	0	0	2	0	1	0
605	2042010	1	0	2	3	1	1	1	1	1	1	0	0	1	1	0	1	1	3	1	1	1
606	2042011	1	1	1	5	1	1	1	1	0	1	0	0	0	0	0	0	0	2	0	1	0
607	2042012	1	0	1	5	1	0	0	0	0	0	0	0	0	0	0	0	0	1	0	0	0
608	2042013	1	1	2	3	2	1	1	1	0	1	0	0	1	0	0	1	0	2	0	1	0
609	2042014	1	0	1	4	1	1	1	1	0	1	0	0	0	1	0	1	0	2	0	1	0
610	2042015	1	1	2	2	2	1	1	1	0	1	0	0	0	1	0	1	0	2	0	1	0
611	2042016	1	0	2	2	1	1	1	1	0	1	0	0	1	1	0	1	1	3	1	1	0
612	2042017	1	0	2	2	3	1	1	1	0	0	0	0	1	0	0	1	0	2	0	1	0
613	2042018	1	1	2	4	2	1	1	1	0	1	0	0	1	1	0	1	1	3	1	1	0
614	2042019	1	1	2	3	2	1	1	1	1	1	0	0	1	1	0	1	1	3	1	1	1
615	2042020	1	0	1	5	1	1	1	1	0	1	0	0	1	0	0	1	0	2	0	1	0
616	2042021	1	1	2	3	2	1	1	1	0	1	1	0	1	1	0	1	1	3	1	1	1
617	2042022	1	1	1	5	1	1	0	1	0	1	0	0	1	1	0	1	1	2	0	1	0
618	2042023	1	1	2	2	2	1	1	1	0	1	0	0	1	1	1	1	1	3	1	1	1
619	2042024	1	1	4	1	2	1	1	1	0	1	0	0	0	0	0	0	0	2	0	1	0
620	2042025	1	1	2	3	1	1	0	1	0	1	0	0	1	1	0	1	1	2	0	1	0
621	205211	1	0	2	3	1	1	1	1	0	0	0	1	0	0	0	1	0	2	0	1	0
622	205212	1	1	2	3	3	1	1	1	0	1	0	1	0	0	1	1	0	3	1	1	0
623	205213	1	0	2	5	1	1	0	1	0	0	0	1	0	0	0	1	0	2	0	1	0
624	205214	1	0	2	3	1	1	1	1	0	1	0	1	0	0	0	1	0	2	0	1	0
625	205215	1	1	4	4	1	1	1	1	0	0	0	1	0	0	0	1	0	2	0	1	0
626	205216	1	0	1	4	1	1	1	1	0	0	0	1	0	0	0	1	0	2	0	1	0
627	205217	1	0	2	2	1	1	1	1	0	1	0	1	0	0	0	1	0	2	0	1	0
628	205218	1	1	4	2	1	1	1	1	1	1	0	1	0	0	1	1	0	3	1	1	1
629	205219	1	1	2	5	1	1	1	1	0	0	0	1	0	0	1	1	0	2	0	1	0
630	2052110	1	0	2	3	2	1	1	1	0	0	0	1	0	0	1	1	0	2	0	1	0
631	2052111	1	0	2	4	1	1	1	1	0	0	0	1	0	0	0	1	0	2	0	1	0
632	2052112	1	1	2	3	2	1	1	1	0	1	0	1	0	0	0	1	0	2	0	1	0
633	2052113	1	0	1	3	2	1	1	1	0	1	0	1	0	0	0	1	0	2	0	1	0
634	2052114	1	0	2	2	1	1	1	1	0	0	0	1	0	0	1	1	0	2	0	1	0
635	2052115	1	0	5	1	2	1	0	1	0	1	0	1	0	0	1	1	0	2	0	1	0
636	2052116	1	1	2	3	1	1	0	1	0	1	0	1	0	0	0	1	0	2	0	1	0
637	2052117	1	1	2	2	2	1	1	1	0	0	0	1	0	0	1	1	0	2	0	1	0
638	2052118	1	0	2	3	1	1	1	1	0	0	0	1	0	0	0	1	0	2	0	1	0
639	2052119	1	0	2	3	1	1	1	1	0	0	0	1	0	0	1	1	0	2	0	1	0
640	2052120	1	1	1	5	1	1	1	1	1	0	0	1	0	0	0	1	0	2	0	1	0
641	2052121	1	0	2	3	2	1	1	1	0	0	0	1	0	0	0	1	0	2	0	1	0
642	2052122	1	1	2	3	1	0	0	0	0	0	0	0	0	0	0	0	0	1	0	0	0
643	2052123	1	0	2	3	2	1	1	1	0	0	0	1	0	0	1	1	0	2	0	1	0
644	2052124	1	0	2	3	1	1	1	1	0	0	0	1	0	0	0	1	0	2	0	1	0
645	2052125	1	1	2	2	1	1	1	1	0	0	0	1	0	0	0	1	0	2	0	1	0
646	2052126	1	1	4	2	2	1	1	1	0	1	0	1	0	0	1	1	0	3	1	1	0
647	2052127	1	0	2	3	1	1	1	1	0	0	0	1	0	0	0	1	0	2	0	1	0
648	2052128	1	0	3	3	2	1	1	1	0	0	0	1	0	0	1	1	0	2	0	1	0
649	2052129	1	1	2	4	2	1	1	1	0	0	0	1	0	0	0	1	0	2	0	1	0
650	2052130	1	0	1	5	1	0	0	0	0	0	0	0	0	0	0	0	0	1	0	0	0
651	205221	1	0	1	2	2	1	1	1	0	1	0	1	0	0	1	1	0	3	1	1	0
652	205222	1	1	1	3	2	1	1	1	0	1	0	1	0	0	0	1	0	2	0	1	0
653	205223	1	1	1	1	1	1	1	1	0	1	0	1	0	0	0	1	0	2	0	1	0
654	205224	1	0	1	1	2	1	1	1	0	1	0	1	0	0	0	1	0	2	0	1	0
655	205225	1	1	3	2	1	1	1	1	0	1	0	1	0	0	0	1	0	2	0	1	0
656	205226	1	1	2	3	1	1	1	1	0	1	0	1	0	0	0	1	0	2	0	1	0
657	205227	1	0	1	4	2	1	1	1	0	1	1	0	0	0	0	1	0	2	0	1	0
658	205228	1	0	1	4	1	1	1	1	1	1	0	1	0	0	0	1	0	3	1	1	0
659	205229	1	0	4	1	2	1	1	1	0	1	0	1	0	0	0	1	0	2	0	1	0

660	2052210	1	1	1	5	2	1	1	1	0	1	0	1	0	0	0	1	0	2	0	1	0
661	2052211	1	0	2	3	2	1	1	1	0	1	0	1	0	0	1	1	0	3	1	1	0
662	2052212	1	0	4	1	2	1	1	1	0	1	0	1	0	0	1	1	0	3	1	1	0
663	2052213	1	0	3	4	2	1	1	1	0	1	0	1	0	0	0	1	0	2	0	1	0
664	2052214	1	0	2	4	2	1	1	1	0	1	0	1	0	0	0	1	0	2	0	1	0
665	2052215	1	0	3	3	2	1	1	1	0	1	0	1	0	0	1	1	0	3	1	1	0
666	2052216	1	0	1	2	1	1	1	1	0	1	0	1	0	0	1	1	0	3	1	1	0
667	2052217	1	0	2	2	1	1	1	1	0	1	0	1	0	0	1	1	0	3	1	1	0
668	2052218	1	0	2	1	1	1	1	1	0	1	0	1	0	0	0	1	0	2	0	1	0
669	2052219	1	0	1	5	1	1	1	1	0	1	0	1	0	0	0	1	0	2	0	1	0
670	2052220	1	0	2	2	2	1	1	1	0	1	0	1	0	0	0	1	0	2	0	1	0
671	2052221	1	0	2	3	2	1	1	1	0	1	0	1	0	0	0	1	0	2	0	1	0
672	2052222	1	0	1	2	3	1	1	1	0	1	0	1	0	0	0	1	0	2	0	1	0
673	2052223	1	0	1	2	1	1	0	1	0	1	1	1	0	0	0	1	1	2	0	1	0
674	2052224	1	1	2	5	2	1	1	1	0	1	0	1	0	0	0	1	0	2	0	1	0
675	2052225	1	0	1	3	2	1	1	1	0	1	0	1	0	0	0	1	0	2	0	1	0
676	2052226	1	0	1	4	2	1	1	1	0	1	0	1	0	0	1	1	0	3	1	1	0
677	2052227	1	0	1	2	1	1	0	1	0	0	0	1	0	0	1	1	0	2	0	1	0
678	2052228	1	0	1	1	1	1	1	1	0	0	0	1	0	0	0	1	0	2	0	1	0
679	2052229	1	0	3	5	2	1	1	1	0	1	0	1	0	0	1	1	0	3	1	1	0
680	2052230	1	0	1	2	1	1	1	1	0	1	0	1	0	0	0	1	0	2	0	1	0
681	205231	1	0	5	1	2	1	1	1	0	1	0	1	0	0	0	1	0	2	0	1	0
682	205232	1	0	1	3	2	1	1	1	0	1	0	1	0	0	0	1	0	2	0	1	0
683	205233	1	0	4	1	2	1	1	1	0	1	0	1	0	0	1	1	0	3	1	1	0
684	205234	1	0	4	3	2	1	1	1	0	1	0	1	0	0	0	1	0	2	0	1	0
685	205235	1	1	3	2	3	1	1	1	0	1	0	0	1	0	0	1	0	2	0	1	0
686	205236	1	0	2	3	2	1	1	1	0	1	0	1	0	0	0	1	0	2	0	1	0
687	205237	1	0	2	2	2	1	1	1	0	1	0	1	0	0	1	1	0	3	1	1	0
688	205238	1	1	1	3	2	1	1	1	0	1	0	1	0	0	0	1	0	2	0	1	0
689	205239	1	0	3	1	1	1	1	1	0	0	0	1	0	0	0	1	0	2	0	1	0
690	2052310	1	1	1	3	2	1	1	1	0	1	0	1	0	0	0	1	0	2	0	1	0
691	2052311	1	1	1	2	1	1	1	1	0	1	0	1	0	0	0	1	0	2	0	1	0
692	2052312	1	1	1	3	2	1	1	1	0	1	0	1	0	0	0	1	0	2	0	1	0
693	2052313	1	0	2	3	2	1	1	1	0	1	0	1	0	0	0	1	0	2	0	1	0
694	2052314	1	0	5	3	2	1	1	1	0	1	0	1	0	0	1	1	0	3	1	1	0
695	2052315	1	0	1	2	2	1	1	1	0	1	0	1	0	0	0	1	0	2	0	1	0
696	2052316	1	1	1	5	2	1	1	1	0	1	0	1	0	0	0	1	0	2	0	1	0
697	2052317	1	0	1	1	2	1	0	1	1	1	0	1	0	0	0	1	0	2	0	1	0
698	2052318	1	1	4	5	3	1	1	1	0	1	0	1	0	0	1	1	0	3	1	1	0
699	2052319	1	1	5	5	3	1	1	1	0	1	0	1	0	0	1	1	0	3	1	1	0
700	2052320	1	0	2	2	3	1	1	1	0	1	0	1	0	0	0	1	0	2	0	1	0
701	2052321	1	1	4	5	2	1	1	1	0	1	0	1	0	0	0	1	0	2	0	1	0
702	2052322	1	0	1	4	2	1	1	1	0	1	0	1	0	0	0	1	0	2	0	1	0
703	2052323	1	1	1	5	2	1	1	1	0	1	1	1	0	0	0	1	1	3	1	1	0
704	2052324	1	0	1	1	1	1	1	1	0	1	0	1	0	0	0	1	0	2	0	1	0
705	2052325	1	1	1	3	2	1	1	1	0	1	0	1	0	0	0	1	0	2	0	1	0
706	2052326	1	0	2	1	2	1	1	1	0	1	0	1	0	0	0	1	0	2	0	1	0
707	2052327	1	1	1	5	1	1	1	1	0	1	0	1	0	0	0	1	0	2	0	1	0
708	2052328	1	1	2	3	2	1	1	1	0	1	0	1	0	0	1	1	0	3	1	1	0
709	2052329	1	1	1	1	2	1	1	1	0	1	0	1	0	0	0	1	0	2	0	1	0
710	2052330	1	0	1	1	1	1	1	0	0	0	0	0	0	0	1	0	0	2	0	1	0
711	205241	1	0	4	1	2	1	1	1	0	1	0	0	0	0	1	0	0	2	0	1	0
712	205242	1	0	2	3	3	1	1	1	0	1	0	0	0	0	1	0	0	2	0	1	0
713	205243	1	0	4	3	3	1	1	1	0	1	0	0	0	1	1	1	0	3	1	1	0
714	205244	1	0	3	3	3	1	1	1	0	1	1	0	0	0	1	1	0	3	1	1	0
715	205245	1	1	4	3	2	1	1	1	0	1	0	1	1	0	1	1	1	3	1	1	1
716	205246	1	0	5	4	3	1	1	1	0	1	0	1	0	1	1	1	1	3	1	1	1
717	205247	1	1	3	4	3	1	1	1	1	1	0	1	0	1	1	1	1	4	1	1	1
718	205248	1	1	2	3	2	1	1	1	0	1	0	1	0	0	0	1	0	2	0	1	0
719	205249	1	0	2	2	2	1	1	1	1	1	0	1	0	0	0	1	0	3	1	1	0

720	2052410	1	0	2	2	3	1	1	0	0	1	0	0	0	0	0	0	0	2	0	1	0
721	2052411	1	1	1	2	3	1	1	1	1	1	0	1	0	0	1	1	0	3	1	1	1
722	2052412	1	0	3	4	3	1	1	1	1	1	0	1	0	0	1	1	0	3	1	1	1
723	2052413	1	1	3	5	2	1	1	1	1	1	0	0	0	1	1	1	0	3	1	1	1
724	2052414	1	1	2	4	3	1	1	1	0	1	0	1	0	0	0	1	0	2	0	1	0
725	2052415	1	0	4	3	3	1	1	1	1	1	0	1	0	0	1	1	0	3	1	1	1
726	2052416	1	1	1	4	2	1	1	1	1	1	0	0	0	0	1	0	0	3	1	1	0
727	2052417	1	0	5	3	3	1	1	1	1	1	0	1	0	1	1	1	1	4	1	1	1
728	2052418	1	1	1	3	2	1	1	0	0	1	0	0	0	0	0	0	0	2	0	1	0
729	2052419	1	0	1	1	2	1	1	1	0	1	0	1	0	0	0	1	0	2	0	1	0
730	2052420	1	0	1	2	3	1	1	1	0	1	0	1	0	0	0	1	0	2	0	1	0
731	2052421	1	1	1	5	2	1	1	1	0	1	0	0	0	0	0	0	0	2	0	1	0
732	2052422	1	1	3	1	3	1	1	1	0	1	0	0	1	0	1	1	0	3	1	1	0
733	2052423	1	0	4	1	2	1	1	1	1	1	0	1	0	0	0	1	0	3	1	1	0
734	2052424	1	0	2	2	2	1	1	1	0	1	0	1	0	0	1	1	0	3	1	1	0
735	2052425	1	1	2	3	3	1	1	1	0	1	0	1	0	0	0	1	0	2	0	1	0
736	2052426	1	1	2	3	3	1	1	1	1	1	0	1	1	0	1	1	1	4	1	1	1
737	2052427	1	0	3	2	3	1	1	1	0	1	0	1	1	0	0	1	1	3	1	1	0
738	2052428	1	1	4	3	3	1	1	1	0	1	0	1	0	0	0	1	0	2	0	1	0
739	2052429	1	0	1	4	3	1	0	1	1	1	0	1	0	0	0	1	0	2	0	1	0
740	2052430	1	0	1	5	2	1	0	1	0	0	0	1	0	0	0	1	0	2	0	1	0
741	205251	1	0	2	2	2	1	1	1	0	1	0	1	0	0	1	1	0	3	1	1	0
742	205252	1	0	1	3	1	1	1	1	0	1	0	1	0	0	0	1	0	2	0	1	0
743	205253	1	0	4	2	2	1	1	1	0	1	0	1	1	0	0	1	1	3	1	1	0
744	205254	1	1	2	5	2	1	1	1	0	1	0	0	0	0	0	0	0	2	0	1	0
745	205255	1	0	1	2	1	1	1	1	0	1	0	1	0	0	0	1	0	2	0	1	0
746	205256	1	0	2	2	2	1	1	1	0	1	0	1	0	0	0	1	0	2	0	1	0
747	205257	1	0	2	2	2	1	1	1	0	1	0	1	0	0	0	1	0	2	0	1	0
748	205258	1	1	1	2	1	1	1	1	0	0	0	1	0	0	0	1	0	2	0	1	0
749	205259	1	0	1	3	1	1	1	1	0	1	0	1	0	0	1	1	0	3	1	1	0
750	2052510	1	1	1	5	1	1	1	1	0	0	0	1	0	0	0	1	0	2	0	1	0
751	2052511	1	1	2	2	1	1	1	1	0	1	0	1	0	0	0	1	0	2	0	1	0
752	2052512	1	0	2	1	1	1	1	1	0	1	0	1	0	0	0	1	0	2	0	1	0
753	2052513	1	0	1	2	1	1	1	1	0	0	0	0	0	0	0	0	0	2	0	1	0
754	2052514	1	0	4	2	2	1	1	1	0	1	0	1	0	0	0	1	0	2	0	1	0
755	2052515	1	0	2	2	2	1	1	1	0	1	0	1	0	0	0	1	0	2	0	1	0
756	2052516	1	0	4	2	2	1	1	1	0	1	0	1	1	0	1	1	1	3	1	1	1
757	2052517	1	0	1	2	1	1	1	1	0	1	0	1	0	0	0	1	0	2	0	1	0
758	2052518	1	0	1	2	1	1	1	1	0	1	0	1	0	0	0	1	0	2	0	1	0
759	2052519	1	1	1	2	1	1	1	1	0	1	0	1	0	0	0	1	0	2	0	1	0
760	2052520	1	0	1	1	2	1	1	1	0	0	0	1	0	0	0	1	0	2	0	1	0
761	2052521	1	0	1	3	1	1	1	1	0	1	0	1	0	0	0	1	0	2	0	1	0
762	2052522	1	0	1	2	2	1	1	1	0	1	0	1	0	0	0	1	0	2	0	1	0
763	2052523	1	0	1	3	2	1	1	1	0	1	0	1	0	0	0	1	0	2	0	1	0
764	2052524	1	0	1	3	2	1	1	1	0	1	0	1	0	0	0	1	0	2	0	1	0
765	2052525	1	0	2	3	3	1	1	1	0	1	0	1	0	0	0	1	0	2	0	1	0
766	2052526	1	0	2	2	1	1	1	1	0	1	0	1	0	0	0	1	0	2	0	1	0
767	2052527	1	0	1	1	2	1	1	1	0	1	0	1	0	0	0	1	0	2	0	1	0
768	2052528	1	1	1	3	2	1	1	1	0	1	0	1	0	0	0	1	0	2	0	1	0
769	2052529	1	0	1	3	1	1	1	1	0	1	0	1	0	0	1	1	0	3	1	1	0
770	2052530	1	1	3	3	2	1	1	1	0	1	0	1	0	0	0	1	0	2	0	1	0
771	206261	1	1	2	3	2	1	1	1	0	1	0	1	0	0	1	1	0	3	1	1	0
772	206262	1	1	2	1	1	1	1	1	0	1	0	1	0	0	0	1	0	2	0	1	0
773	206263	1	1	3	3	2	1	0	1	0	1	0	1	0	0	1	1	0	2	0	1	0
774	206264	1	1	4	2	2	1	1	1	0	1	0	1	0	1	1	1	1	3	1	1	1
775	206265	1	1	2	4	2	1	1	1	0	1	0	1	0	0	1	1	0	3	1	1	0
776	206266	1	0	2	1	1	1	0	1	0	1	0	1	0	0	0	1	0	2	0	1	0
777	206267	1	1	3	2	2	1	0	1	0	1	0	0	0	1	1	1	0	2	0	1	0
778	206268	1	0	4	1	2	1	0	1	0	0	0	1	0	0	1	1	0	2	0	1	0
779	206269	1	0	2	4	2	1	1	1	0	0	0	1	0	0	1	1	0	2	0	1	0

780	2062610	1	0	2	1	2	1	0	1	0	1	0	1	0	0	0	1	0	2	0	1	0
781	2062611	1	0	1	2	1	1	1	1	0	1	0	1	0	0	1	1	0	3	1	1	0
782	2062612	1	0	4	1	1	1	1	1	0	1	0	1	0	1	1	1	1	3	1	1	1
783	2062613	1	0	1	3	2	1	0	1	0	1	0	1	0	0	0	1	0	2	0	1	0
784	2062614	1	0	2	3	2	1	1	1	1	1	0	1	0	0	1	1	0	3	1	1	1
785	2062615	1	1	1	4	1	1	0	0	0	0	0	0	0	0	0	0	0	2	0	0	0
786	2062616	1	1	1	3	1	1	1	1	0	0	0	1	0	0	0	1	0	2	0	1	0
787	2062617	1	0	3	1	1	1	1	1	0	0	0	1	0	0	0	1	0	2	0	1	0
788	2062618	1	1	1	5	2	1	1	1	1	1	0	1	1	0	0	1	1	3	1	1	1
789	2062619	1	0	2	2	2	1	0	1	0	1	0	1	0	1	0	1	1	2	0	1	0
790	2062620	1	0	1	2	2	1	1	1	0	0	0	1	0	0	0	1	0	2	0	1	0
791	2062621	1	0	1	2	1	1	0	1	0	1	0	0	0	0	1	0	0	2	0	1	0
792	2062622	1	0	1	2	3	1	1	1	0	0	0	1	0	0	1	1	0	2	0	1	0
793	2062623	1	0	1	4	2	1	1	1	0	0	0	0	0	0	0	0	0	2	0	1	0
794	2062624	1	1	2	3	2	1	1	1	0	1	0	1	0	1	1	1	1	3	1	1	1
795	2062625	1	1	3	3	2	1	0	1	0	0	0	1	0	0	0	1	0	2	0	1	0
796	206271	1	0	1	4	1	1	0	1	0	0	0	1	1	0	0	1	1	2	0	1	0
797	206272	1	0	4	1	3	1	1	1	0	1	0	1	0	0	0	1	0	2	0	1	0
798	206273	1	0	2	2	2	1	0	1	0	0	0	1	0	0	0	1	0	2	0	1	0
799	206274	1	0	2	1	3	1	0	1	0	0	0	0	0	0	0	0	0	2	0	1	0
800	206275	1	1	1	1	1	1	0	1	0	0	0	0	0	0	0	0	0	2	0	1	0
801	206276	1	0	2	3	2	1	1	1	0	0	0	1	1	0	0	1	1	2	0	1	0
802	206277	1	1	4	1	2	1	0	1	0	1	0	1	0	0	1	1	0	2	0	1	0
803	206278	1	0	4	2	2	1	1	1	0	1	0	1	0	1	0	1	1	3	1	1	0
804	206279	1	1	2	3	1	1	0	1	0	1	0	1	0	1	1	1	1	3	1	1	1
805	2062710	1	0	2	2	1	1	0	1	0	1	0	1	0	1	1	1	1	3	1	1	1
806	2062711	1	0	1	3	1	1	0	1	0	1	0	1	1	1	0	1	1	3	1	1	1
807	2062712	1	0	1	2	2	1	0	1	0	0	0	1	0	0	1	1	0	2	0	1	0
808	2062713	1	1	3	1	2	1	0	1	1	1	0	0	0	0	1	0	0	2	0	1	0
809	2062714	1	1	2	1	2	1	0	1	0	0	0	0	0	0	0	0	0	2	0	1	0
810	2062715	1	1	1	1	2	1	0	0	0	0	0	0	0	0	1	0	0	2	0	0	0
811	2062716	1	1	3	2	1	1	0	1	0	1	0	0	0	0	1	0	0	2	0	1	0
812	2062717	1	0	3	1	2	1	1	1	0	1	0	1	0	0	0	1	0	2	0	1	0
813	2062718	1	1	1	1	1	1	0	0	0	0	0	0	0	0	0	0	0	2	0	0	0
814	2062719	1	0	2	2	2	1	0	1	0	1	0	1	0	1	0	1	1	2	0	1	0
815	2062720	1	0	3	1	2	1	0	1	0	1	0	1	0	0	0	1	0	2	0	1	0
816	2062721	1	1	1	4	1	1	1	1	0	0	0	1	0	0	0	1	0	2	0	1	0
817	2062722	1	0	3	2	1	1	1	1	0	0	0	1	0	0	1	1	0	2	0	1	0
818	2062723	1	1	2	3	1	1	0	1	1	1	0	1	0	1	1	1	1	3	1	1	1
819	2062724	1	0	2	2	2	1	1	1	0	0	0	1	0	0	1	1	0	2	0	1	0
820	2062725	1	1	2	2	2	1	0	1	0	1	0	1	0	1	1	1	1	3	1	1	1
821	2062726	1	1	1	1	1	1	0	1	0	1	0	1	1	1	0	1	1	3	1	1	1
822	2062727	1	1	1	3	2	1	0	0	0	0	0	0	0	0	0	0	0	2	0	0	0
823	2062728	1	0	2	1	2	1	0	1	0	0	0	0	0	0	0	0	0	2	0	1	0
824	2062729	1	0	1	3	2	1	0	1	0	1	0	1	0	1	0	1	1	2	0	1	0
825	2062730	1	0	2	2	1	1	0	1	0	1	0	1	0	1	0	1	1	2	0	1	0
826	206281	1	0	3	3	2	1	0	1	0	0	0	1	0	0	0	1	0	2	0	1	0
827	206282	1	1	4	2	2	1	1	1	0	0	0	1	0	0	0	1	0	2	0	1	0
828	206283	1	1	2	3	2	1	1	1	1	0	0	1	0	0	1	1	0	3	1	1	0
829	206284	1	1	2	3	2	1	1	1	1	0	0	0	0	0	0	0	0	2	0	1	0
830	206285	1	0	2	2	2	1	1	1	0	0	0	1	1	0	0	1	1	2	0	1	0
831	206286	1	0	2	5	2	1	1	1	0	0	0	1	0	0	0	1	0	2	0	1	0
832	206287	1	0	2	2	2	1	1	1	0	1	0	0	1	0	0	1	0	2	0	1	0
833	206288	1	0	1	2	2	1	1	1	0	0	0	1	0	0	0	1	0	2	0	1	0
834	206289	1	0	4	1	2	1	0	1	0	0	0	1	0	0	0	1	0	2	0	1	0
835	2062810	1	1	1	2	2	1	1	1	0	0	0	0	0	0	0	0	0	2	0	1	0
836	2062811	1	1	1	4	1	1	1	1	0	0	0	1	0	0	0	1	0	2	0	1	0
837	2062812	1	1	1	2	1	1	1	1	0	0	0	1	0	0	0	1	0	2	0	1	0
838	2062813	1	0	3	1	1	1	0	1	0	0	0	1	0	0	0	1	0	2	0	1	0
839	2062814	1	1	4	4	2	1	1	1	1	0	0	1	1	1	0	1	1	3	1	1	1

840	2062815	1	0	2	3	1	1	0	1	0	0	0	1	0	0	0	1	0	2	0	1	0
841	2062816	1	1	2	1	2	1	1	1	0	0	0	0	0	1	0	1	0	2	0	1	0
842	2062817	1	0	2	4	2	1	0	1	0	0	0	1	0	0	1	1	0	2	0	1	0
843	2062818	1	0	1	3	2	1	1	1	1	0	0	1	0	0	0	1	0	2	0	1	0
844	2062819	1	0	2	4	2	1	0	1	1	0	0	1	0	0	0	1	0	2	0	1	0
845	2062820	1	1	1	4	3	1	1	1	0	0	0	1	0	1	0	1	1	2	0	1	0
846	2062821	1	0	1	3	2	1	1	1	0	1	0	1	0	1	0	1	1	3	1	1	0
847	2062822	1	1	2	3	3	1	0	1	1	1	1	1	1	0	1	1	1	4	1	1	1
848	2062823	1	1	1	3	3	1	1	1	1	0	1	1	0	0	0	1	1	3	1	1	0
849	2062824	1	0	1	3	2	1	1	1	1	0	0	1	0	0	1	1	0	3	1	1	0
850	2062825	1	0	2	2	2	1	1	1	0	0	0	1	0	0	1	1	0	2	0	1	0
851	2062826	1	1	1	3	1	1	0	1	0	0	0	1	0	0	0	1	0	2	0	1	0
852	2062827	1	1	4	3	3	1	0	1	0	0	0	1	0	0	0	1	0	2	0	1	0
853	2062828	1	1	2	5	2	1	1	1	1	1	0	1	1	0	0	1	1	3	1	1	1
854	2062829	1	1	1	5	2	1	1	1	0	0	0	1	0	0	0	1	0	2	0	1	0
855	2062830	1	1	4	4	3	1	1	1	1	0	1	1	0	0	1	1	1	3	1	1	1
856	206291	1	1	1	5	2	1	1	1	0	0	1	1	0	1	0	1	1	3	1	1	0
857	206292	1	0	2	3	2	1	1	1	0	0	0	1	0	1	1	1	1	3	1	1	0
858	206293	1	0	1	3	2	1	1	1	0	1	0	1	0	1	1	1	1	3	1	1	1
859	206294	1	1	2	2	2	1	1	1	0	1	0	1	0	1	0	1	1	3	1	1	0
860	206295	1	1	1	5	2	1	0	1	0	0	0	1	0	1	0	1	1	2	0	1	0
861	206296	1	0	1	4	2	1	1	1	0	0	1	1	0	0	0	1	1	2	0	1	0
862	206297	1	1	1	5	2	1	1	1	0	0	1	1	0	1	0	1	1	3	1	1	0
863	206298	1	1	1	5	1	0	0	0	0	0	0	0	0	0	0	0	0	1	0	0	0
864	206299	1	0	2	4	3	1	1	1	1	1	1	1	1	0	1	1	1	4	1	1	1
865	2062910	1	1	1	5	3	0	0	0	0	0	0	0	0	0	0	0	0	1	0	0	0
866	2062911	1	0	2	2	2	1	0	1	1	1	0	1	0	1	0	1	1	3	1	1	1
867	2062912	1	0	1	3	2	1	0	1	0	0	0	1	0	0	0	1	0	2	0	1	0
868	2062913	1	0	1	2	2	1	1	1	0	0	0	1	1	0	1	1	1	3	1	1	0
869	2062914	1	0	1	3	2	1	1	1	0	1	1	1	0	0	0	1	1	3	1	1	0
870	2062915	1	1	5	1	2	1	1	1	0	1	1	1	0	1	1	1	1	4	1	1	1
871	2062916	1	1	4	2	3	1	1	1	1	0	0	1	1	0	0	1	1	3	1	1	0
872	2062917	1	1	1	5	2	1	0	1	0	1	0	1	0	0	1	1	0	2	0	1	0
873	2062918	1	0	3	2	2	1	0	1	0	1	0	1	0	0	0	1	0	2	0	1	0
874	2062919	1	0	1	3	2	1	1	1	0	1	0	1	0	0	0	1	0	2	0	1	0
875	2062920	1	0	4	1	3	1	0	1	1	1	0	1	1	0	0	1	1	3	1	1	1
876	2062921	1	1	2	3	3	1	0	1	0	0	0	1	0	0	1	1	0	2	0	1	0
877	2062922	1	0	1	2	2	1	1	1	0	0	0	0	1	1	0	1	1	2	0	1	0
878	2062923	1	0	4	3	2	1	1	1	1	1	0	1	0	0	0	1	0	3	1	1	0
879	2062924	1	1	1	4	3	1	1	1	0	0	1	1	0	0	0	1	1	2	0	1	0
880	2062925	1	1	1	2	2	1	0	1	0	1	0	1	0	0	0	1	0	2	0	1	0
881	2062926	1	0	2	2	2	1	0	1	1	1	0	1	0	0	0	1	0	2	0	1	0
882	2062927	1	0	2	2	2	1	0	1	1	1	0	1	1	0	1	1	1	3	1	1	1
883	2062928	1	0	1	2	2	1	0	1	0	1	0	1	0	0	1	1	0	2	0	1	0
884	2062929	1	0	1	2	2	1	0	1	0	0	0	0	0	0	0	0	0	2	0	1	0
885	2062930	1	1	2	4	2	1	1	0	0	0	0	0	0	0	0	0	0	2	0	1	0
886	206301	1	0	2	5	2	1	1	1	0	1	0	1	1	0	1	1	1	3	1	1	1
887	206302	1	0	1	4	2	1	1	1	1	1	0	1	1	0	1	1	1	4	1	1	1
888	206303	1	0	4	5	1	0	0	0	0	0	0	0	0	0	0	0	0	1	0	0	0
889	206304	1	0	4	1	2	1	1	1	0	0	0	1	1	1	1	1	1	3	1	1	1
890	206305	1	0	1	5	2	0	0	0	0	0	0	0	0	0	0	0	0	1	0	0	0
891	206306	1	1	3	4	2	1	1	1	0	1	1	1	1	1	1	1	1	4	1	1	1
892	206307	1	1	3	3	2	1	1	1	1	1	0	1	1	0	1	1	1	4	1	1	1
893	206308	1	0	1	5	2	0	0	0	0	0	0	0	0	0	1	0	0	2	0	0	0
894	206309	1	0	5	2	2	1	1	1	0	1	0	1	1	1	1	1	1	4	1	1	1
895	2063010	1	1	1	3	3	1	1	1	0	0	0	1	1	1	1	1	1	3	1	1	1
896	2063011	1	1	2	4	3	1	1	1	1	0	0	1	1	1	1	1	1	4	1	1	1
897	2063012	1	0	2	2	2	1	1	1	0	1	0	1	1	0	1	1	1	3	1	1	1
898	2063013	1	0	5	1	3	1	1	1	0	0	0	1	1	0	1	1	1	3	1	1	0
899	2063014	1	0	4	5	3	1	1	1	1	0	0	1	1	1	1	1	1	4	1	1	1

900	2063015	1	1	1	5	2	0	0	0	0	0	0	0	0	0	1	0	0	2	0	0	0
901	2063016	1	0	2	3	2	1	1	1	0	1	0	1	1	1	1	1	1	4	1	1	1
902	2063017	1	1	3	4	3	1	1	1	1	0	0	1	1	1	1	1	1	4	1	1	1
903	2063018	1	0	4	3	2	1	1	1	0	1	0	1	1	1	1	1	1	4	1	1	1
904	2063019	1	0	2	3	1	1	1	1	0	1	0	1	0	0	1	1	0	3	1	1	0
905	2063020	1	0	2	2	3	1	1	1	0	0	1	1	0	1	1	1	1	3	1	1	1
906	2063021	1	0	2	1	2	1	1	1	0	1	0	1	1	0	1	1	1	3	1	1	1
907	2063022	1	1	2	2	2	1	1	1	1	1	0	1	1	0	1	1	1	4	1	1	1
908	2063023	1	1	2	2	2	1	0	1	0	1	0	1	1	1	1	1	1	3	1	1	1
909	2063024	1	1	2	3	2	1	0	1	1	1	0	0	1	0	1	1	0	3	1	1	1
910	2063025	1	0	2	5	2	0	0	0	0	0	0	0	0	0	0	0	0	1	0	0	0
911	206311	1	0	2	1	2	1	1	1	0	0	0	1	1	0	1	1	1	3	1	1	0
912	206312	1	0	3	2	2	1	1	1	0	0	0	1	1	1	1	1	1	3	1	1	1
913	206313	1	1	2	2	2	0	0	0	0	0	0	0	0	0	0	0	0	1	0	0	0
914	206314	1	1	1	3	2	1	1	1	1	0	0	1	1	1	1	1	1	4	1	1	1
915	206315	1	0	2	2	2	1	1	1	0	0	0	1	1	0	1	1	1	3	1	1	0
916	206316	1	0	5	1	3	1	1	1	0	1	0	1	0	0	1	1	0	3	1	1	0
917	206317	1	0	1	2	2	1	1	1	1	1	0	1	1	1	1	1	1	4	1	1	1
918	206318	1	1	1	3	2	1	1	1	1	0	0	1	1	0	1	1	1	3	1	1	1
919	206319	1	0	1	5	1	0	0	0	0	0	0	0	0	0	0	0	0	1	0	0	0
920	2063110	1	1	2	2	2	1	1	1	1	1	0	1	0	1	1	1	1	4	1	1	1
921	2063111	1	0	1	3	2	1	1	1	0	1	0	1	1	1	1	1	1	4	1	1	1
922	2063112	1	0	1	2	2	1	1	1	0	0	0	1	0	0	1	1	0	2	0	1	0
923	2063113	1	1	2	2	2	1	1	1	0	0	0	1	1	1	1	1	1	3	1	1	1
924	2063114	1	1	2	3	2	1	1	1	1	1	0	1	1	0	1	1	1	4	1	1	1
925	2063115	1	0	2	3	2	0	0	0	0	0	0	0	0	0	1	0	0	2	0	0	0
926	2063116	1	1	2	2	2	1	1	1	1	1	0	1	1	0	1	1	1	4	1	1	1
927	2063117	1	0	3	3	2	1	1	1	0	1	0	1	0	1	1	1	1	3	1	1	1
928	2063118	1	1	2	3	2	1	1	1	1	1	0	1	1	0	0	1	1	3	1	1	1
929	2063119	1	0	2	2	2	1	1	1	0	0	0	1	1	0	1	1	1	3	1	1	0
930	2063120	1	1	2	1	2	1	1	1	1	1	0	1	1	0	1	1	1	4	1	1	1
931	2063121	1	1	2	2	2	1	1	1	0	0	0	1	0	1	1	1	1	3	1	1	0
932	2063122	1	1	1	4	2	1	1	1	0	1	0	1	1	1	1	1	1	4	1	1	1
933	2063123	1	0	1	2	2	1	1	1	1	1	0	1	0	1	1	1	1	4	1	1	1
934	2063124	1	0	1	1	3	1	1	1	0	0	0	1	1	1	1	1	1	3	1	1	1
935	2063125	1	1	2	2	3	1	1	1	0	0	0	1	1	1	1	1	1	3	1	1	1
936	206321	1	0	1	2	1	1	1	1	0	0	0	1	0	0	0	1	0	2	0	1	0
937	206322	1	0	1	2	1	1	1	1	0	1	0	1	0	0	0	1	0	2	0	1	0
938	206323	1	0	2	1	2	1	0	1	0	1	0	1	0	1	0	1	1	2	0	1	0
939	206324	1	1	2	4	1	1	1	1	0	0	0	1	0	1	0	1	1	2	0	1	0
940	206325	1	0	3	1	2	1	1	0	0	0	0	0	0	0	0	0	0	2	0	1	0
941	206326	1	0	1	2	1	1	0	1	0	0	0	1	0	0	0	1	0	2	0	1	0
942	206327	1	0	1	5	1	1	1	1	0	0	0	1	0	0	0	1	0	2	0	1	0
943	206328	1	0	1	3	1	1	1	1	0	1	0	1	0	0	0	1	0	2	0	1	0
944	206329	1	0	3	1	3	1	1	1	1	0	1	0	0	1	0	1	1	3	1	1	0
945	2063210	1	0	3	1	1	1	1	1	0	1	1	0	1	1	0	1	1	3	1	1	1
946	2063211	1	1	1	4	1	1	1	1	0	0	1	0	1	0	0	1	1	2	0	1	0
947	2063212	1	1	2	3	2	1	0	0	0	1	0	0	0	0	0	0	0	2	0	0	0
948	2063213	1	0	1	3	2	1	1	1	0	1	0	1	1	1	0	1	1	3	1	1	1
949	2063214	1	0	2	2	2	1	1	1	0	1	0	1	0	0	0	1	0	2	0	1	0
950	2063215	1	0	3	1	2	1	1	1	0	1	0	1	0	0	0	1	0	2	0	1	0
951	2063216	1	0	1	4	2	1	1	1	0	0	0	1	0	0	0	1	0	2	0	1	0
952	2063217	1	1	2	2	2	1	0	1	0	1	0	1	1	1	1	1	1	3	1	1	1
953	2063218	1	1	4	1	2	1	0	1	0	1	1	0	0	0	0	1	0	2	0	1	0
954	2063219	1	0	2	5	2	1	0	0	0	0	0	0	0	0	0	0	0	2	0	0	0
955	2063220	1	0	1	2	2	1	0	0	0	1	0	0	0	0	0	0	0	2	0	0	0
956	2063221	1	1	2	2	2	1	1	1	0	1	0	1	0	0	0	1	0	2	0	1	0
957	2063222	1	0	2	3	3	1	0	1	0	1	0	1	0	0	0	1	0	2	0	1	0
958	2063223	1	0	2	2	2	1	0	1	0	1	0	1	0	0	0	1	0	2	0	1	0
959	2063224	1	0	1	5	2	1	1	1	0	1	0	0	0	0	0	0	0	2	0	1	0

960	2063225	1	0	1	3	1	1	1	1	0	0	0	0	0	0	0	0	0	2	0	1	0
961	2063226	1	1	2	2	1	1	0	1	0	1	0	1	0	1	0	1	1	2	0	1	0
962	2063227	1	1	3	3	2	1	0	1	0	1	0	1	0	1	0	1	1	2	0	1	0
963	2063228	1	1	3	1	2	1	0	1	0	1	0	0	0	0	0	0	0	2	0	1	0
964	2063229	1	0	4	1	3	1	1	1	0	1	0	1	0	1	1	1	1	3	1	1	1
965	2063230	1	1	1	4	1	1	1	0	0	0	0	0	0	0	0	0	0	2	0	1	0
966	206331	1	1	1	3	1	1	1	1	0	0	0	0	0	0	0	0	0	2	0	1	0
967	206332	1	1	1	3	1	1	1	1	0	0	0	1	0	0	0	1	0	2	0	1	0
968	206333	1	0	2	2	1	1	1	1	0	0	0	1	1	1	0	1	1	3	1	1	0
969	206334	1	1	3	5	1	1	1	1	0	1	0	0	0	0	0	0	0	2	0	1	0
970	206335	1	1	2	3	1	1	1	1	1	1	0	1	0	0	1	1	0	3	1	1	1
971	206336	1	1	2	2	2	1	1	1	0	0	0	1	0	0	1	1	0	2	0	1	0
972	206337	1	0	1	3	1	1	1	1	0	1	0	1	0	0	1	1	0	3	1	1	0
973	206338	1	0	2	4	1	1	1	1	0	0	0	1	1	0	1	1	1	3	1	1	0
974	206339	1	1	2	3	2	1	1	1	0	0	0	1	0	1	1	1	1	3	1	1	0
975	2063310	1	0	1	2	1	1	1	1	0	1	0	1	0	0	0	1	0	2	0	1	0
976	2063311	1	1	2	5	1	1	1	1	0	0	0	1	0	0	0	1	0	2	0	1	0
977	2063312	1	1	1	2	1	1	1	1	0	0	0	0	0	0	0	0	0	2	0	1	0
978	2063313	1	1	2	2	2	1	1	1	0	0	0	0	0	0	0	0	0	2	0	1	0
979	2063314	1	0	2	4	1	1	1	1	0	1	0	1	0	0	1	1	0	3	1	1	0
980	2063315	1	1	2	4	2	1	1	1	0	0	0	1	0	0	1	1	0	2	0	1	0
981	2063316	1	0	2	1	1	1	0	1	1	0	0	0	0	0	0	0	0	2	0	1	0
982	2063317	1	0	1	2	1	1	1	1	0	1	0	1	0	0	1	1	0	3	1	1	0
983	2063318	1	0	1	4	1	1	1	1	0	1	0	1	0	0	1	1	0	3	1	1	0
984	2063319	1	1	1	2	1	1	1	1	0	0	0	1	0	0	1	1	0	2	0	1	0
985	2063320	1	1	2	3	2	1	1	1	0	1	0	1	0	1	0	1	1	3	1	1	0
986	2063321	1	0	4	3	1	1	1	1	0	0	0	0	0	1	1	1	0	2	0	1	0
987	2063322	1	0	2	1	1	1	1	1	0	0	0	1	0	0	0	1	0	2	0	1	0
988	2063323	1	1	4	2	2	1	1	1	0	1	0	1	0	1	0	1	1	3	1	1	0
989	2063324	1	1	2	4	1	1	1	1	0	0	0	1	0	0	0	1	0	2	0	1	0
990	2063325	1	0	2	5	1	1	1	1	1	0	0	1	0	0	0	1	0	2	0	1	0
991	2063326	1	1	1	5	1	1	1	1	0	0	0	0	0	0	0	0	0	2	0	1	0
992	2063327	1	0	2	5	1	1	1	1	0	1	0	1	0	1	1	1	1	3	1	1	1
993	2063328	1	1	1	3	1	1	1	1	0	1	0	1	0	1	1	1	1	3	1	1	1
994	2063329	1	0	1	2	2	1	1	1	0	1	0	0	0	0	0	0	0	2	0	1	0
995	2063330	1	0	2	1	1	1	1	1	0	0	0	0	0	0	1	0	0	2	0	1	0
996	307341	2	1	4	5	3	1	1	1	0	0	1	0	1	0	1	1	1	3	1	1	0
997	307342	2	1	5	5	2	1	1	1	0	1	0	0	1	0	1	1	0	3	1	1	0
998	307343	2	0	3	2	1	1	1	1	0	0	1	0	1	0	1	1	1	3	1	1	0
999	307344	2	1	2	4	2	1	1	1	0	0	1	0	0	1	1	1	1	3	1	1	0
1000	307345	2	1	3	4	2	1	1	1	0	0	1	0	1	1	1	1	1	3	1	1	1
1001	307346	2	1	5	4	2	1	1	1	0	0	1	0	1	0	1	1	1	3	1	1	0
1002	307347	2	0	4	3	2	1	1	1	0	0	1	0	0	1	1	1	1	3	1	1	0
1003	307348	2	1	4	2	1	1	1	1	0	1	1	0	0	1	1	1	1	3	1	1	1
1004	307349	2	1	3	3	2	1	1	1	0	0	1	0	0	1	1	1	1	3	1	1	0
1005	3073410	2	0	3	2	1	1	1	1	1	0	1	0	0	1	1	1	1	3	1	1	1
1006	3073411	2	1	3	1	2	1	1	1	0	0	1	0	1	0	1	1	1	3	1	1	0
1007	3073412	2	0	5	1	3	1	1	1	0	0	1	0	0	1	1	1	1	3	1	1	0
1008	3073413	2	0	2	1	1	1	0	1	0	0	1	0	0	1	1	1	1	2	0	1	0
1009	3073414	2	1	3	5	2	0	0	0	0	0	0	0	0	0	1	0	0	2	0	0	0
1010	3073415	2	1	2	4	1	1	1	1	0	1	1	0	0	1	1	1	1	3	1	1	1
1011	3073416	2	1	3	2	1	1	1	1	0	0	1	0	0	1	1	1	1	3	1	1	0
1012	3073417	2	1	3	2	1	1	1	1	0	0	1	0	0	1	1	1	1	3	1	1	0
1013	3073418	2	1	2	4	1	0	0	0	0	0	0	0	0	0	1	0	0	2	0	0	0
1014	3073419	2	1	3	2	1	1	0	1	0	0	1	0	0	1	1	1	1	2	0	1	0
1015	3073420	2	1	2	2	1	1	1	1	0	0	1	0	1	0	1	1	1	3	1	1	0
1016	3073421	2	1	2	3	2	1	1	1	0	0	1	0	1	0	1	1	1	3	1	1	0
1017	3073422	2	1	2	3	2	1	1	1	0	0	1	0	1	0	1	1	1	3	1	1	0
1018	3073423	2	1	4	4	2	1	0	1	0	0	1	0	1	0	1	1	1	2	0	1	0
1019	3073424	2	1	5	2	3	1	1	1	0	0	1	0	1	1	1	1	1	3	1	1	1

1020	3073425	2	1	2	3	1	1	1	1	0	0	1	0	1	0	1	1	1	3	1	1	0
1021	3073426	2	1	2	4	1	1	1	1	0	0	1	0	1	0	1	1	1	3	1	1	0
1022	3073427	2	1	2	4	1	1	0	1	1	1	1	0	1	0	1	1	1	3	1	1	1
1023	3073428	2	1	5	1	3	1	1	1	0	1	1	0	0	1	1	1	1	3	1	1	1
1024	3073429	2	1	4	2	2	1	1	1	1	0	1	0	1	0	1	1	1	3	1	1	1
1025	3073430	2	1	4	4	1	0	0	0	0	0	0	0	0	0	1	0	0	2	0	0	0
1026	3073431	2	1	2	4	1	1	1	1	1	0	1	0	0	1	1	1	1	3	1	1	1
1027	3073432	2	1	5	2	2	1	1	1	0	0	1	0	1	0	1	1	1	3	1	1	0
1028	307351	2	0	5	2	3	1	1	1	1	1	1	0	0	1	1	1	1	4	1	1	1
1029	307352	2	1	2	2	1	1	1	1	1	1	1	0	0	1	1	1	1	4	1	1	1
1030	307353	2	1	2	2	1	1	0	1	0	0	1	0	0	1	1	1	1	2	0	1	0
1031	307354	2	0	2	4	1	1	1	1	1	1	1	0	0	1	1	1	1	4	1	1	1
1032	307355	2	1	3	3	2	1	1	1	1	1	1	1	0	1	1	1	1	4	1	1	1
1033	307356	2	0	2	2	1	1	1	1	0	0	1	0	0	1	1	1	1	3	1	1	0
1034	307357	2	0	2	5	1	1	1	1	0	0	1	0	0	1	1	1	1	3	1	1	0
1035	307358	2	0	2	4	1	1	1	1	0	0	1	0	0	1	1	1	1	3	1	1	0
1036	307359	2	0	3	2	1	1	1	1	0	1	1	0	0	1	1	1	1	3	1	1	1
1037	3073510	2	1	3	2	1	1	1	1	0	1	1	0	0	1	1	1	1	3	1	1	1
1038	3073511	2	1	2	5	2	1	1	1	1	1	1	0	0	1	1	1	1	4	1	1	1
1039	3073512	2	0	4	1	2	1	1	1	0	1	1	1	0	1	1	1	1	4	1	1	1
1040	3073513	2	1	2	3	1	1	1	1	0	0	1	0	0	1	1	1	1	3	1	1	0
1041	3073514	2	1	3	3	1	1	1	1	1	1	1	0	0	1	1	1	1	4	1	1	1
1042	3073515	2	0	2	3	1	1	1	1	1	1	1	0	0	1	1	1	1	4	1	1	1
1043	3073516	2	0	2	3	1	1	1	1	1	1	1	0	0	1	1	1	1	4	1	1	1
1044	3073517	2	0	2	4	1	1	1	1	0	0	1	0	0	1	1	1	1	3	1	1	0
1045	3073518	2	0	3	2	1	1	1	1	0	0	1	0	0	0	1	1	0	2	0	1	0
1046	3073519	2	0	3	2	1	1	1	1	0	0	1	0	0	0	1	1	0	2	0	1	0
1047	3073520	2	1	2	2	1	1	1	1	1	0	1	0	0	1	1	1	1	3	1	1	1
1048	3073521	2	0	5	2	2	1	1	1	0	0	1	0	0	1	1	1	1	3	1	1	0
1049	3073522	2	1	5	2	2	1	1	1	1	0	1	0	0	1	1	1	1	3	1	1	1
1050	3073523	2	0	3	3	2	0	0	0	0	0	0	0	0	0	1	0	0	2	0	0	0
1051	3073524	2	1	5	3	3	1	1	1	1	0	1	0	0	1	1	1	1	3	1	1	1
1052	3073525	2	1	5	2	3	1	1	1	1	0	1	0	0	1	1	1	1	3	1	1	1
1053	3073526	2	1	4	4	3	1	1	1	1	1	1	0	1	0	1	1	1	4	1	1	1
1054	3073527	2	1	5	1	2	1	1	1	0	0	1	0	0	1	1	1	1	3	1	1	0
1055	3073528	2	1	4	3	3	1	1	1	0	0	1	0	0	1	1	1	1	3	1	1	0
1056	3073529	2	1	5	2	3	1	1	1	0	1	1	0	0	1	1	1	1	3	1	1	1
1057	3073530	2	1	2	2	2	1	1	1	0	1	1	0	0	1	1	1	1	3	1	1	1
1058	3073531	2	1	3	2	2	1	1	1	0	0	1	0	1	1	1	1	1	3	1	1	1
1059	3073532	2	1	4	5	2	1	0	1	0	0	0	0	1	1	1	1	1	2	0	1	0
1060	3073533	2	1	5	2	2	1	1	1	1	0	1	0	1	0	1	1	1	3	1	1	1
1061	307361	2	1	4	2	2	1	1	1	1	1	1	1	0	0	1	1	1	4	1	1	1
1062	307362	2	1	5	2	3	1	1	1	1	1	1	0	1	0	1	1	1	4	1	1	1
1063	307363	2	1	5	5	2	1	0	1	0	1	0	1	0	1	0	1	1	2	0	1	0
1064	307364	2	1	2	5	2	1	1	1	1	1	1	1	0	1	1	1	1	4	1	1	1
1065	307365	2	0	3	3	2	1	1	1	1	1	1	1	0	0	1	1	1	4	1	1	1
1066	307366	2	1	4	4	2	1	1	1	1	1	0	1	0	1	0	1	1	3	1	1	1
1067	307367	2	1	2	5	1	1	1	1	1	1	1	0	0	0	1	1	0	3	1	1	1
1068	307368	2	1	5	4	3	1	1	1	1	1	0	1	1	1	1	1	1	4	1	1	1
1069	307369	2	0	5	4	3	1	1	1	1	1	0	1	1	1	1	1	1	4	1	1	1
1070	3073610	2	0	2	2	2	1	0	1	1	1	0	1	0	0	0	1	0	2	0	1	0
1071	3073611	2	0	2	4	2	1	1	1	1	1	1	1	0	1	1	1	1	4	1	1	1
1072	3073612	2	1	5	3	3	1	1	1	1	1	1	1	1	1	1	1	1	4	1	1	1
1073	3073613	2	0	4	2	2	1	1	1	1	1	0	1	1	1	0	1	1	4	1	1	1
1074	3073614	2	1	2	5	1	1	1	1	1	1	1	1	1	1	0	1	1	4	1	1	1
1075	3073615	2	0	4	3	3	1	0	1	1	1	1	1	0	1	1	1	1	4	1	1	1
1076	3073616	2	1	2	4	1	1	1	1	1	1	0	1	0	1	0	1	1	3	1	1	1
1077	3073617	2	1	5	4	3	1	1	1	1	1	0	1	1	1	1	1	1	4	1	1	1
1078	3073618	2	1	4	2	3	1	1	1	1	1	0	1	1	1	0	1	1	4	1	1	1
1079	3073619	2	1	2	5	2	1	1	1	1	1	0	1	0	0	0	1	0	3	1	1	0

1080	3073620	2	1	2	2	2	1	1	1	1	1	1	1	1	1	1	1	1	4	1	1	1
1081	3073621	2	1	4	2	2	1	1	1	1	1	0	1	1	0	0	1	1	3	1	1	1
1082	3073622	2	0	3	3	2	1	1	1	1	1	0	1	1	0	0	1	1	3	1	1	1
1083	3073623	2	1	4	5	2	1	1	1	0	1	0	1	0	0	0	1	0	2	0	1	0
1084	3073624	2	1	3	4	1	1	1	1	1	1	0	1	1	0	0	1	1	3	1	1	1
1085	3073625	2	0	2	5	2	1	1	1	1	1	0	1	1	0	0	1	1	3	1	1	1
1086	3073626	2	1	4	4	2	1	0	1	1	1	0	1	0	0	0	1	0	2	0	1	0
1087	3073627	2	0	2	3	1	1	1	1	1	1	1	0	1	1	1	1	1	4	1	1	1
1088	3073628	2	1	2	5	2	1	1	1	0	1	0	1	0	0	0	1	0	2	0	1	0
1089	3073629	2	1	3	2	2	1	0	1	1	1	0	1	1	0	0	1	1	3	1	1	1
1090	3073630	2	1	3	4	1	1	1	1	1	1	0	1	1	0	0	1	1	3	1	1	1
1091	307371	2	0	4	1	2	1	0	1	0	0	0	0	0	0	1	0	0	2	0	1	0
1092	307372	2	0	4	2	2	1	1	1	1	1	0	1	1	0	1	1	1	4	1	1	1
1093	307373	2	0	2	2	2	1	0	1	0	1	0	0	0	0	1	0	0	2	0	1	0
1094	307374	2	0	2	3	2	0	0	0	0	0	0	0	0	0	0	0	0	1	0	0	0
1095	307375	2	0	2	2	1	1	1	1	1	1	0	1	1	0	0	1	1	3	1	1	1
1096	307376	2	0	4	1	1	1	0	1	1	1	0	1	1	1	0	1	1	3	1	1	1
1097	307377	2	1	2	2	2	1	0	1	1	1	1	1	0	1	1	1	1	4	1	1	1
1098	307378	2	0	2	5	2	0	0	0	0	0	0	0	0	0	0	0	0	1	0	0	0
1099	307379	2	1	2	2	2	1	1	1	1	1	0	1	0	1	1	1	1	4	1	1	1
1100	3073710	2	0	3	3	2	1	1	1	1	1	0	1	0	1	1	1	1	4	1	1	1
1101	3073711	2	1	2	1	1	0	0	0	0	0	0	0	0	0	0	0	0	1	0	0	0
1102	3073712	2	1	1	5	1	0	0	0	0	0	0	0	0	0	0	0	0	1	0	0	0
1103	3073713	2	1	2	1	1	1	0	1	0	1	0	0	0	0	0	0	0	2	0	1	0
1104	3073714	2	1	4	4	2	1	0	1	1	1	1	1	0	0	0	1	1	3	1	1	1
1105	3073715	2	0	3	2	3	1	1	1	1	1	1	0	1	1	1	1	1	4	1	1	1
1106	3073716	2	1	2	2	2	1	1	1	1	1	0	0	0	0	1	0	0	3	1	1	0
1107	3073717	2	0	3	1	1	1	0	1	0	0	0	0	0	0	0	0	0	2	0	1	0
1108	3073718	2	0	3	2	2	0	0	0	0	0	0	0	0	0	1	0	0	2	0	0	0
1109	3073719	2	1	2	5	1	0	0	0	0	0	0	0	0	0	1	0	0	2	0	0	0
1110	3073720	2	0	3	2	1	1	0	1	0	0	0	0	1	1	0	1	1	2	0	1	0
1111	3073721	2	1	2	5	2	1	0	1	0	0	0	0	0	0	0	0	0	2	0	1	0
1112	3073722	2	0	2	2	1	1	0	1	0	0	0	0	0	0	0	0	0	2	0	1	0
1113	3073723	2	0	2	3	2	1	1	1	1	1	0	1	0	1	1	1	1	4	1	1	1
1114	3073724	2	0	2	1	2	1	1	1	1	1	1	1	1	0	1	1	1	4	1	1	1
1115	3073725	2	0	3	4	2	1	0	1	0	0	0	0	0	0	1	0	0	2	0	1	0
1116	3073726	2	1	2	2	2	0	0	0	0	0	0	0	0	0	1	0	0	2	0	0	0
1117	3073727	2	1	2	2	2	1	1	1	1	1	0	1	0	0	1	1	0	3	1	1	1
1118	3073728	2	0	2	1	1	0	0	0	0	0	0	0	0	0	0	0	0	1	0	0	0
1119	3073729	2	0	3	2	2	0	0	0	0	0	0	0	0	0	1	0	0	2	0	0	0
1120	3073730	2	0	2	1	1	0	0	0	0	0	0	0	0	0	1	0	0	2	0	0	0
1121	307381	2	0	2	2	2	1	1	1	1	1	1	0	0	1	1	1	1	4	1	1	1
1122	307382	2	1	3	4	2	1	1	1	1	1	1	0	0	1	1	1	1	4	1	1	1
1123	307383	2	0	3	2	2	1	1	1	1	1	1	0	0	1	1	1	1	4	1	1	1
1124	307384	2	1	2	3	2	1	1	1	1	1	1	0	0	0	1	1	0	3	1	1	1
1125	307385	2	0	4	1	2	1	1	1	1	1	1	0	1	1	1	1	1	4	1	1	1
1126	307386	2	1	2	3	2	1	1	1	1	1	1	1	0	1	1	1	1	4	1	1	1
1127	307387	2	0	3	1	2	1	1	1	1	0	1	0	1	1	1	1	1	4	1	1	1
1128	307388	2	1	3	3	2	1	1	1	1	1	1	0	0	1	0	1	1	3	1	1	1
1129	307389	2	1	4	3	2	1	1	1	1	1	1	0	0	1	0	1	1	3	1	1	1
1130	3073810	2	0	4	2	2	1	1	1	1	1	1	0	0	1	1	1	1	4	1	1	1
1131	3073811	2	0	5	2	2	1	1	1	1	1	1	0	0	1	1	1	1	4	1	1	1
1132	3073812	2	1	3	2	2	1	1	1	1	1	1	0	0	1	1	1	1	4	1	1	1
1133	3073813	2	0	2	3	2	1	1	1	1	1	1	0	0	0	1	1	0	3	1	1	1
1134	3073814	2	1	5	2	2	1	1	1	1	1	1	0	1	1	0	1	1	4	1	1	1
1135	3073815	2	0	5	2	3	1	1	1	1	1	1	0	0	1	0	1	1	3	1	1	1
1136	3073816	2	1	2	2	1	1	1	1	1	1	1	0	1	1	1	1	1	4	1	1	1
1137	3073817	2	0	5	2	3	1	1	1	1	1	1	0	1	1	1	1	1	4	1	1	1
1138	3073818	2	1	3	4	2	1	1	1	1	1	1	0	1	1	1	1	1	4	1	1	1
1139	3073819	2	0	4	1	3	1	1	1	1	1	1	0	1	1	1	1	1	4	1	1	1

1140	3073820	2	1	4	3	2	1	1	1	1	1	1	0	0	1	0	1	1	3	1	1	1
1141	3073821	2	0	5	2	2	1	1	1	1	1	1	0	1	1	1	1	1	4	1	1	1
1142	3073822	2	1	2	4	2	1	1	1	1	1	1	1	1	1	1	1	1	4	1	1	1
1143	3073823	2	0	3	2	2	1	1	1	1	1	1	0	1	1	1	1	1	4	1	1	1
1144	3073824	2	1	2	4	2	1	1	1	1	1	1	0	0	1	1	1	1	4	1	1	1
1145	3073825	2	0	4	1	2	1	1	1	1	1	1	0	1	1	1	1	1	4	1	1	1
1146	3073826	2	0	2	4	2	1	1	1	1	1	1	1	1	1	1	1	1	4	1	1	1
1147	3073827	2	1	2	4	2	1	1	1	1	1	1	1	0	1	1	1	1	4	1	1	1
1148	3073828	2	1	4	4	3	1	1	1	1	1	1	0	1	1	1	1	1	4	1	1	1
1149	3073829	2	0	2	2	2	1	1	1	0	1	1	0	1	1	1	1	1	4	1	1	1
1150	3073830	2	1	2	4	2	1	1	1	1	1	1	1	1	0	1	1	1	4	1	1	1
1151	307391	2	1	4	3	2	1	1	1	1	1	1	0	0	1	1	1	1	4	1	1	1
1152	307392	2	1	4	1	2	1	1	1	1	1	1	0	0	1	1	1	1	4	1	1	1
1153	307393	2	1	2	2	2	1	1	1	1	1	1	0	0	1	0	1	1	3	1	1	1
1154	307394	2	0	2	2	2	0	0	0	0	0	0	0	0	0	0	0	0	1	0	0	0
1155	307395	2	1	4	1	2	0	0	0	0	0	0	0	0	0	1	0	0	2	0	0	0
1156	307396	2	0	2	2	2	0	0	0	0	0	0	0	0	0	1	0	0	2	0	0	0
1157	307397	2	1	2	3	2	0	0	0	0	0	0	0	0	0	1	0	0	2	0	0	0
1158	307398	2	1	3	4	2	0	0	0	0	0	0	0	0	0	0	0	0	1	0	0	0
1159	307399	2	0	3	4	1	1	0	1	0	0	0	0	0	0	0	0	0	2	0	1	0
1160	3073910	2	1	2	4	2	1	1	1	1	1	1	0	1	1	1	1	1	4	1	1	1
1161	3073911	2	1	3	5	1	1	1	1	1	0	0	0	0	0	1	0	0	2	0	1	0
1162	3073912	2	0	5	1	2	1	1	1	1	0	1	0	1	1	1	1	1	4	1	1	1
1163	3073913	2	1	5	3	2	1	1	1	0	1	1	0	1	1	1	1	1	4	1	1	1
1164	3073914	2	0	2	2	1	0	0	0	0	0	0	0	0	0	0	0	0	1	0	0	0
1165	3073915	2	0	3	1	2	0	0	0	0	0	0	0	0	0	0	0	0	1	0	0	0
1166	3073916	2	0	2	2	1	0	0	0	0	0	0	0	0	0	0	0	0	1	0	0	0
1167	3073917	2	0	2	2	1	0	0	0	0	0	0	0	0	0	0	0	0	1	0	0	0
1168	3073918	2	1	2	4	1	0	0	0	0	0	0	0	0	0	0	0	0	1	0	0	0
1169	3073919	2	1	2	4	1	0	0	0	0	0	0	0	0	0	0	0	0	1	0	0	0
1170	3073920	2	0	2	2	2	0	0	0	0	0	0	0	0	0	0	0	0	1	0	0	0
1171	3073921	2	1	2	2	1	1	1	1	1	1	1	0	0	1	0	1	1	3	1	1	1
1172	3073922	2	1	2	5	1	1	1	1	1	1	1	0	0	1	0	1	1	3	1	1	1
1173	3073923	2	1	2	2	1	0	0	0	0	0	0	0	0	0	0	0	0	1	0	0	0
1174	3073924	2	0	2	2	2	0	0	0	0	0	0	0	0	0	1	0	0	2	0	0	0
1175	3073925	2	1	2	5	1	0	0	0	0	0	0	0	0	0	1	0	0	2	0	0	0
1176	3073926	2	0	2	2	1	1	1	1	0	1	1	0	0	1	0	1	1	3	1	1	0
1177	3073927	2	0	2	2	2	1	1	1	1	1	1	0	0	1	0	1	1	3	1	1	1
1178	3073928	2	0	2	3	2	0	0	0	0	0	0	0	0	0	0	0	0	1	0	0	0
1179	3073929	2	0	2	3	1	0	0	0	0	0	0	0	0	0	1	0	0	2	0	0	0
1180	3073930	2	1	4	2	2	0	0	0	0	0	0	0	0	0	0	0	0	1	0	0	0
1181	307401	2	0	3	3	2	1	1	1	1	1	1	0	0	0	0	1	0	3	1	1	0
1182	307402	2	1	2	3	1	1	1	1	0	1	1	1	0	0	0	1	1	3	1	1	0
1183	307403	2	0	2	2	1	1	1	1	1	0	1	1	0	1	1	1	1	4	1	1	1
1184	307404	2	1	3	3	2	1	1	1	1	1	1	1	0	0	1	1	1	4	1	1	1
1185	307405	2	1	2	3	2	1	1	1	0	1	1	0	0	1	1	1	1	3	1	1	1
1186	307406	2	1	3	3	2	1	1	1	1	1	1	1	0	0	1	1	1	4	1	1	1
1187	307407	2	0	2	2	2	1	1	1	1	0	1	1	0	0	0	1	1	3	1	1	0
1188	307408	2	0	4	1	2	1	1	1	1	1	1	1	0	0	0	1	1	3	1	1	1
1189	307409	2	0	3	3	2	1	1	1	1	1	1	0	0	1	1	1	1	4	1	1	1
1190	3074010	2	1	5	1	2	1	1	1	1	1	1	0	0	1	1	1	1	4	1	1	1
1191	3074011	2	1	2	3	1	1	0	1	1	1	1	0	0	1	0	1	1	3	1	1	1
1192	3074012	2	1	5	2	3	1	1	1	1	1	1	0	0	1	0	1	1	3	1	1	1
1193	3074013	2	0	4	1	2	1	1	1	1	1	1	0	1	1	0	1	1	4	1	1	1
1194	3074014	2	0	3	2	2	1	1	1	1	1	1	0	1	1	1	1	1	4	1	1	1
1195	3074015	2	0	4	2	2	1	1	1	1	1	1	1	0	1	1	1	1	4	1	1	1
1196	3074016	2	1	5	2	3	1	1	1	1	1	1	1	0	1	1	1	1	4	1	1	1
1197	3074017	2	0	2	2	1	1	1	1	1	1	1	1	0	1	1	1	1	4	1	1	1
1198	3074018	2	1	2	2	1	1	1	1	1	1	1	1	0	1	1	1	1	4	1	1	1
1199	3074019	2	1	3	1	1	1	1	1	1	1	1	0	0	1	1	1	1	4	1	1	1

1200	3074020	2	1	2	1	1	1	1	1	1	1	1	0	0	1	1	1	1	4	1	1	1
1201	3074021	2	0	3	2	2	1	1	1	1	1	1	1	0	1	1	1	1	4	1	1	1
1202	3074022	2	0	2	2	1	1	1	1	1	1	1	0	0	1	1	1	1	4	1	1	1
1203	3074023	2	0	3	2	2	1	1	1	1	1	1	1	0	1	1	1	1	4	1	1	1
1204	3074024	2	1	2	5	2	1	1	1	1	1	1	1	1	1	1	1	1	4	1	1	1
1205	3074025	2	0	2	3	1	1	1	1	1	1	1	0	1	1	1	1	1	4	1	1	1
1206	3074026	2	0	4	2	2	1	1	1	1	1	1	0	0	1	0	1	1	3	1	1	1
1207	3074027	2	1	3	1	2	1	1	1	1	0	1	0	0	1	1	1	1	3	1	1	1
1208	3074028	2	0	2	3	2	1	1	1	1	0	1	0	0	1	1	1	1	3	1	1	1
1209	3074029	2	1	3	2	2	1	1	1	1	0	1	1	0	1	1	1	1	4	1	1	1
1210	3074030	2	1	3	3	1	1	1	1	1	1	1	0	0	1	1	1	1	4	1	1	1
1211	3074031	2	1	2	2	2	1	1	1	1	1	1	0	0	1	1	1	1	4	1	1	1
1212	3074032	2	0	4	1	2	1	1	1	1	0	1	1	0	1	1	1	1	4	1	1	1
1213	3074033	2	0	5	1	2	1	1	1	0	1	1	0	0	1	1	1	1	3	1	1	1
1214	3074034	2	1	4	4	1	1	1	1	0	1	1	0	0	1	0	1	1	3	1	1	0
1215	3074035	2	0	5	2	2	1	1	1	1	1	1	0	0	1	0	1	1	3	1	1	1
1216	307411	2	0	2	2	2	1	1	1	1	1	0	1	1	1	1	1	1	4	1	1	1
1217	307412	2	0	2	4	2	1	1	1	0	1	0	1	1	1	1	1	1	4	1	1	1
1218	307413	2	0	2	3	2	1	1	1	1	1	0	1	1	1	1	1	1	4	1	1	1
1219	307414	2	0	4	1	1	1	1	1	1	1	0	1	1	1	1	1	1	4	1	1	1
1220	307415	2	0	2	2	2	1	1	1	1	1	0	1	1	0	1	1	1	4	1	1	1
1221	307416	2	1	2	3	2	1	1	1	1	1	1	1	1	1	0	1	1	4	1	1	1
1222	307417	2	0	2	1	2	1	1	1	1	1	0	1	1	0	1	1	1	4	1	1	1
1223	307418	2	0	4	2	2	1	1	1	1	1	0	1	1	1	0	1	1	4	1	1	1
1224	307419	2	0	5	2	2	1	1	1	1	1	1	1	1	1	0	1	1	4	1	1	1
1225	3074110	2	0	2	2	2	1	1	1	1	1	1	1	1	1	0	1	1	4	1	1	1
1226	3074111	2	1	3	1	2	1	1	1	1	1	0	1	1	1	0	1	1	4	1	1	1
1227	3074112	2	0	4	2	1	1	1	1	1	1	1	1	1	1	0	1	1	4	1	1	1
1228	3074113	2	0	2	2	2	1	1	1	1	1	1	1	1	0	1	1	1	4	1	1	1
1229	3074114	2	1	3	2	2	1	1	1	1	1	1	1	0	1	1	1	1	4	1	1	1
1230	3074115	2	1	3	3	2	1	1	1	1	1	1	1	0	1	1	1	1	4	1	1	1
1231	3074116	2	1	2	3	3	1	1	1	1	1	1	1	1	1	1	1	1	4	1	1	1
1232	3074117	2	1	2	3	2	1	1	1	1	1	0	1	1	1	1	1	1	4	1	1	1
1233	3074118	2	0	3	3	2	1	1	1	1	1	1	0	0	0	1	1	0	3	1	1	1
1234	3074119	2	0	5	4	2	1	1	1	1	1	1	1	0	1	1	1	1	4	1	1	1
1235	3074120	2	1	5	5	3	1	1	1	1	1	0	1	1	1	1	1	1	4	1	1	1
1236	3074121	2	1	3	4	2	1	1	1	1	1	1	1	1	1	1	1	1	4	1	1	1
1237	3074122	2	1	3	3	2	1	1	1	1	1	1	1	1	0	1	1	1	4	1	1	1
1238	3074123	2	0	3	4	2	1	1	1	0	1	1	0	0	0	1	1	0	3	1	1	0
1239	3074124	2	0	2	5	1	1	1	1	1	1	1	1	1	1	1	1	1	4	1	1	1
1240	3074125	2	1	2	5	2	1	1	1	1	1	1	1	1	1	1	1	1	4	1	1	1
1241	3074126	2	0	5	2	2	1	1	1	1	1	1	0	1	1	1	1	1	4	1	1	1
1242	3074127	2	0	2	5	2	1	1	1	1	1	0	1	1	1	1	1	1	4	1	1	1
1243	3074128	2	0	2	5	2	1	1	1	1	1	0	1	1	1	1	1	1	4	1	1	1
1244	3074129	2	1	5	4	2	1	1	1	1	1	1	0	1	0	0	1	1	3	1	1	1
1245	3074130	2	0	3	2	3	1	1	1	1	1	0	1	1	0	1	1	1	4	1	1	1
1246	3074131	2	1	2	5	2	1	1	1	1	1	1	0	1	1	0	1	1	4	1	1	1
1247	3074132	2	0	2	2	2	0	0	0	0	0	0	0	0	0	0	0	0	1	0	0	0
1248	3074133	2	1	2	4	2	0	0	0	0	0	0	0	0	0	1	0	0	2	0	0	0
1249	3074134	2	0	5	2	3	1	1	1	1	1	0	1	1	1	1	1	1	4	1	1	1
1250	3074135	2	1	2	5	2	1	1	1	0	1	0	1	1	1	0	1	1	3	1	1	1
1251	3074136	2	1	2	5	2	1	1	1	1	1	1	1	1	0	1	1	1	4	1	1	1
1252	3074137	2	1	2	5	2	0	0	0	0	0	0	0	0	0	0	0	0	1	0	0	0
1253	3074138	2	0	3	3	2	1	1	1	1	1	0	0	0	0	0	0	0	2	0	1	0
1254	3074139	2	1	2	3	2	0	0	0	0	0	0	0	0	0	0	0	0	1	0	0	0
1255	3074140	2	1	2	5	2	0	0	0	0	0	0	0	0	0	0	0	0	1	0	0	0
1256	308421	2	0	2	4	1	0	0	0	0	0	0	0	0	0	0	0	0	1	0	0	0
1257	308422	2	1	2	4	2	1	1	1	0	1	1	0	1	1	0	1	1	3	1	1	1
1258	308423	2	0	3	3	3	1	1	1	0	0	0	1	1	1	0	1	1	3	1	1	0
1259	308424	2	0	2	2	1	1	1	1	1	1	0	1	0	1	0	1	1	3	1	1	1

1260	308425	2	0	2	3	3	1	1	1	0	0	0	1	1	1	1	1	1	3	1	1	1
1261	308426	2	0	2	2	3	1	1	1	0	0	0	0	1	1	1	1	1	3	1	1	0
1262	308427	2	1	2	2	1	0	0	0	0	0	0	0	0	0	0	0	0	1	0	0	0
1263	308428	2	1	2	4	2	1	0	1	0	1	0	0	1	1	0	1	1	2	0	1	0
1264	308429	2	0	4	1	2	1	1	1	0	0	0	0	1	1	0	1	1	2	0	1	0
1265	3084210	2	0	2	2	2	0	0	0	0	0	0	0	0	0	1	0	0	2	0	0	0
1266	3084211	2	1	2	3	1	0	0	0	0	0	0	0	0	0	0	0	0	1	0	0	0
1267	3084212	2	0	1	3	2	0	0	0	0	0	0	0	0	0	0	0	0	1	0	0	0
1268	3084213	2	0	2	2	2	0	0	0	0	0	0	0	0	0	0	0	0	1	0	0	0
1269	3084214	2	0	2	2	2	0	0	0	0	0	0	0	0	0	0	0	0	1	0	0	0
1270	3084215	2	1	2	2	1	0	0	0	0	0	0	0	0	0	0	0	0	1	0	0	0
1271	3084216	2	1	2	3	2	0	0	0	0	0	0	0	0	0	0	0	0	1	0	0	0
1272	3084217	2	1	2	2	2	0	0	0	0	0	0	0	0	0	0	0	0	1	0	0	0
1273	3084218	2	1	2	2	2	1	1	1	0	0	1	0	1	1	0	1	1	3	1	1	0
1274	3084219	2	1	3	3	2	1	1	1	0	0	1	0	0	1	1	1	1	3	1	1	0
1275	3084220	2	1	4	4	3	0	0	0	0	0	0	0	0	0	1	0	0	2	0	0	0
1276	3084221	2	0	2	2	2	1	1	1	1	0	1	0	1	1	1	1	1	4	1	1	1
1277	3084222	2	0	3	4	2	0	0	0	0	0	0	0	0	0	0	0	0	1	0	0	0
1278	3084223	2	0	2	4	1	1	0	1	1	0	0	0	0	0	1	0	0	2	0	1	0
1279	3084224	2	0	3	2	2	0	0	0	0	0	0	0	0	0	0	0	0	1	0	0	0
1280	3084225	2	1	2	3	2	0	0	0	0	0	0	0	0	0	1	0	0	2	0	0	0
1281	3084226	2	0	4	2	3	1	0	1	0	0	0	1	1	1	1	1	1	3	1	1	1
1282	3084227	2	1	2	4	2	0	0	0	0	0	0	0	0	0	0	0	0	1	0	0	0
1283	3084228	2	1	1	5	2	0	0	0	0	0	0	0	0	0	1	0	0	2	0	0	0
1284	3084229	2	0	3	1	3	1	1	0	0	0	0	0	0	0	1	0	0	2	0	1	0
1285	3084230	2	1	2	4	2	0	0	0	0	0	0	0	0	0	1	0	0	2	0	0	0
1286	308431	2	0	2	3	2	1	1	1	1	0	1	0	0	0	1	1	0	3	1	1	0
1287	308432	2	1	4	5	2	1	1	1	1	0	1	0	0	0	0	1	0	2	0	1	0
1288	308433	2	0	2	2	1	1	1	1	0	0	0	0	0	0	0	0	0	2	0	1	0
1289	308434	2	0	2	4	2	0	0	0	0	0	0	0	0	0	1	0	0	2	0	0	0
1290	308435	2	0	4	2	1	1	1	1	0	0	0	0	0	0	1	0	0	2	0	1	0
1291	308436	2	1	4	1	2	1	1	1	0	0	1	0	0	0	0	1	0	2	0	1	0
1292	308437	2	0	5	1	2	0	0	0	0	0	0	0	0	0	0	0	0	1	0	0	0
1293	308438	2	1	2	2	2	0	0	0	0	0	0	0	0	0	0	0	0	1	0	0	0
1294	308439	2	0	2	2	2	0	0	0	0	0	0	0	0	0	1	0	0	2	0	0	0
1295	3084310	2	1	3	3	1	1	1	1	1	0	0	1	0	1	1	1	1	3	1	1	1
1296	3084311	2	0	2	2	2	1	1	1	0	0	0	0	0	0	0	0	0	2	0	1	0
1297	3084312	2	0	2	2	2	0	0	0	0	0	0	0	0	0	0	0	0	1	0	0	0
1298	3084313	2	1	2	5	2	0	0	0	0	0	0	0	0	0	0	0	0	1	0	0	0
1299	3084314	2	0	4	1	2	0	0	0	0	0	0	0	0	0	1	0	0	2	0	0	0
1300	3084315	2	1	4	4	3	0	0	0	0	0	0	0	0	0	1	0	0	2	0	0	0
1301	3084316	2	1	2	1	3	1	1	1	1	0	1	0	0	0	1	1	0	3	1	1	0
1302	3084317	2	1	4	1	2	1	1	0	0	0	0	0	0	0	1	0	0	2	0	1	0
1303	3084318	2	0	2	1	2	0	0	0	0	0	0	0	0	0	0	0	0	1	0	0	0
1304	3084319	2	0	2	2	2	0	0	0	0	0	0	0	0	0	1	0	0	2	0	0	0
1305	3084320	2	1	3	2	3	0	0	0	0	0	0	0	0	0	0	0	0	1	0	0	0
1306	3084321	2	1	2	5	2	1	1	1	0	0	0	0	0	0	0	0	0	2	0	1	0
1307	3084322	2	0	2	3	3	0	0	0	0	0	0	0	0	0	0	0	0	1	0	0	0
1308	3084323	2	0	2	3	2	0	0	0	0	0	0	0	0	0	1	0	0	2	0	0	0
1309	3084324	2	1	2	1	3	0	0	0	0	0	0	0	0	0	0	0	0	1	0	0	0
1310	3084325	2	0	4	3	3	0	0	0	0	0	0	0	0	0	0	0	0	1	0	0	0
1311	3084326	2	0	2	1	1	0	0	0	0	0	0	0	0	0	0	0	0	1	0	0	0
1312	3084327	2	0	2	1	1	0	0	0	0	0	0	0	0	0	1	0	0	2	0	0	0
1313	3084328	2	0	5	1	2	0	0	0	0	0	0	0	0	0	0	0	0	1	0	0	0
1314	3084329	2	1	5	5	3	1	1	1	0	0	0	0	0	1	0	1	0	2	0	1	0
1315	3084330	2	0	2	2	2	0	0	0	0	0	0	0	0	0	0	0	0	1	0	0	0
1316	3084331	2	1	4	4	2	1	1	1	0	0	0	0	1	1	0	1	1	2	0	1	0
1317	3084332	2	1	5	2	2	1	1	1	0	0	1	0	0	1	1	1	1	3	1	1	0
1318	3084333	2	0	5	2	3	0	0	0	0	0	0	0	0	0	1	0	0	2	0	0	0
1319	3084334	2	1	5	4	3	0	0	0	0	0	0	0	0	0	1	0	0	2	0	0	0

1320	3084335	2	1	5	4	3	1	1	1	0	0	1	0	0	0	1	1	0	2	0	1	0
1321	3084336	2	1	5	4	3	1	1	1	0	0	0	0	1	1	1	1	1	3	1	1	0
1322	3084337	2	0	2	4	3	1	0	1	0	0	1	0	1	1	1	1	1	3	1	1	1
1323	3084338	2	1	4	3	3	0	0	0	0	0	0	0	0	0	1	0	0	2	0	0	0
1324	3084339	2	1	3	4	3	1	1	1	0	0	0	0	0	1	0	1	0	2	0	1	0
1325	308441	2	0	3	2	2	1	1	1	0	0	0	0	0	0	0	0	0	2	0	1	0
1326	308442	2	0	2	2	2	0	0	0	0	0	0	0	0	0	0	0	0	1	0	0	0
1327	308443	2	0	2	2	2	0	0	0	0	0	0	0	0	0	1	0	0	2	0	0	0
1328	308444	2	0	2	5	2	1	0	1	0	0	0	0	0	0	1	0	0	2	0	1	0
1329	308445	2	0	2	5	2	1	0	1	0	0	0	0	0	0	0	0	0	2	0	1	0
1330	308446	2	1	2	4	2	1	0	1	0	0	0	1	0	0	0	1	0	2	0	1	0
1331	308447	2	1	2	5	2	0	0	0	0	0	0	0	0	0	0	0	0	1	0	0	0
1332	308448	2	1	2	5	2	0	0	0	0	0	0	0	0	0	0	0	0	1	0	0	0
1333	308449	2	0	2	1	1	1	0	1	0	0	0	0	0	0	0	0	0	2	0	1	0
1334	3084410	2	1	3	3	2	0	0	0	0	0	0	0	0	0	0	0	0	1	0	0	0
1335	3084411	2	1	1	5	2	0	0	0	0	0	0	0	0	0	0	0	0	1	0	0	0
1336	3084412	2	1	4	3	3	1	0	1	0	0	0	0	0	0	0	0	0	2	0	1	0
1337	3084413	2	1	2	5	2	1	1	1	0	1	0	1	0	0	0	1	0	2	0	1	0
1338	3084414	2	1	4	3	3	1	0	1	0	0	1	0	0	0	1	1	0	2	0	1	0
1339	3084415	2	1	2	1	3	1	0	1	0	0	0	1	0	0	0	1	0	2	0	1	0
1340	3084416	2	0	2	3	2	0	0	0	0	0	0	0	0	0	0	0	0	1	0	0	0
1341	3084417	2	0	5	1	3	1	1	1	0	1	0	0	0	0	0	0	0	2	0	1	0
1342	3084418	2	1	3	2	3	0	0	0	0	0	0	0	0	0	1	0	0	2	0	0	0
1343	3084419	2	1	4	1	2	1	1	1	0	0	0	0	0	0	0	0	0	2	0	1	0
1344	3084420	2	1	2	5	1	0	0	0	0	0	0	0	0	0	0	0	0	1	0	0	0
1345	3084421	2	0	3	2	3	0	0	0	0	0	0	0	0	0	1	0	0	2	0	0	0
1346	3084422	2	0	2	2	2	0	0	0	0	0	0	0	0	0	1	0	0	2	0	0	0
1347	3084423	2	1	5	2	3	1	1	1	0	0	0	0	0	0	1	0	0	2	0	1	0
1348	3084424	2	1	5	1	3	1	1	1	0	1	0	0	0	0	1	0	0	2	0	1	0
1349	3084425	2	1	4	2	3	0	0	0	0	0	0	0	0	0	0	0	0	1	0	0	0
1350	3084426	2	0	5	1	3	1	1	1	0	0	0	0	0	1	1	1	0	2	0	1	0
1351	308451	2	0	1	4	2	1	0	1	1	0	1	0	0	1	1	1	1	3	1	1	1
1352	308452	2	1	2	2	1	1	1	1	1	0	0	0	0	1	1	1	0	3	1	1	0
1353	308453	2	0	2	3	2	0	0	0	0	0	0	0	0	0	1	0	0	2	0	0	0
1354	308454	2	0	2	2	1	0	0	0	0	0	0	0	0	0	1	0	0	2	0	0	0
1355	308455	2	0	1	4	1	1	1	0	1	0	0	0	0	0	1	0	0	2	0	1	0
1356	308456	2	1	2	2	2	1	1	1	1	0	0	0	0	1	1	1	0	3	1	1	0
1357	308457	2	1	2	2	1	1	1	1	1	0	0	0	0	1	1	1	0	3	1	1	0
1358	308458	2	0	2	3	2	0	0	0	0	0	0	0	0	0	0	0	0	1	0	0	0
1359	308459	2	0	1	2	1	1	1	1	1	0	0	0	0	1	1	1	0	3	1	1	0
1360	3084510	2	0	2	2	2	1	1	1	1	0	0	0	0	1	1	1	0	3	1	1	0
1361	3084511	2	1	1	4	1	1	1	1	0	0	1	0	0	1	1	1	1	3	1	1	0
1362	3084512	2	0	2	2	1	1	1	1	0	1	0	0	0	1	1	1	0	3	1	1	0
1363	3084513	2	0	2	1	1	1	1	1	1	1	1	0	0	1	1	1	1	4	1	1	1
1364	3084514	2	0	2	1	1	1	1	1	0	0	0	0	1	1	1	1	1	3	1	1	0
1365	3084515	2	1	1	4	1	1	1	1	1	0	0	0	0	1	1	1	0	3	1	1	0
1366	3084516	2	1	2	4	1	1	0	1	1	0	0	0	0	0	1	0	0	2	0	1	0
1367	3084517	2	0	2	4	1	1	0	1	1	0	0	0	0	1	0	1	0	2	0	1	0
1368	3084518	2	1	2	3	1	1	1	1	1	0	0	0	0	0	1	0	0	2	0	1	0
1369	3084519	2	0	1	4	1	1	1	1	0	0	0	1	0	0	1	1	0	2	0	1	0
1370	3084520	2	0	2	5	1	1	1	1	1	1	0	0	0	1	1	1	0	3	1	1	1
1371	3084521	2	0	2	4	1	1	1	1	1	0	0	0	0	1	1	1	0	3	1	1	0
1372	3084522	2	0	2	2	2	1	1	0	1	0	0	0	0	0	1	0	0	2	0	1	0
1373	3084523	2	0	2	1	1	1	1	1	1	1	0	0	0	1	1	1	0	3	1	1	1
1374	3084524	2	1	2	4	1	1	0	1	0	0	1	0	0	0	1	1	0	2	0	1	0
1375	3084525	2	1	2	5	1	1	0	1	1	1	1	0	0	1	1	1	1	3	1	1	1
1376	308461	2	0	3	4	3	1	1	1	1	1	1	0	1	1	1	1	1	4	1	1	1
1377	308462	2	1	1	3	1	1	1	1	1	1	1	0	0	0	1	1	0	3	1	1	1
1378	308463	2	1	2	2	2	1	1	0	0	0	0	0	0	0	1	0	0	2	0	1	0
1379	308464	2	1	1	4	1	1	1	0	1	0	0	0	0	0	1	0	0	2	0	1	0

1380	308465	2	0	2	5	1	1	1	1	0	0	0	0	0	0	1	0	0	2	0	1	0
1381	308466	2	1	2	3	1	1	1	0	1	0	0	0	0	0	0	0	0	2	0	1	0
1382	308467	2	0	2	4	2	1	1	1	0	1	0	0	0	0	1	0	0	2	0	1	0
1383	308468	2	0	2	3	2	1	0	0	0	0	0	0	0	0	1	0	0	2	0	0	0
1384	308469	2	0	2	5	1	1	1	0	0	0	0	0	0	0	1	0	0	2	0	1	0
1385	3084610	2	1	2	2	1	1	0	0	1	0	0	0	0	0	1	0	0	2	0	0	0
1386	3084611	2	1	2	2	1	1	1	0	1	0	0	0	0	0	1	0	0	2	0	1	0
1387	3084612	2	0	2	3	2	1	1	0	1	0	0	0	0	0	1	0	0	2	0	1	0
1388	3084613	2	0	4	3	2	1	1	1	1	0	0	0	0	1	1	1	0	3	1	1	0
1389	3084614	2	1	2	5	1	1	1	1	1	0	0	0	0	0	1	0	0	2	0	1	0
1390	3084615	2	1	2	5	2	1	1	0	1	1	0	0	0	0	1	0	0	2	0	1	0
1391	3084616	2	1	4	4	1	1	1	1	1	1	0	0	0	1	1	1	0	3	1	1	1
1392	3084617	2	0	2	4	1	1	1	0	1	1	0	0	0	0	1	0	0	2	0	1	0
1393	3084618	2	1	2	4	1	1	1	0	0	1	0	0	0	0	1	0	0	2	0	1	0
1394	3084619	2	1	2	4	1	1	1	0	0	1	0	0	0	0	1	0	0	2	0	1	0
1395	3084620	2	1	2	3	1	1	1	0	1	1	0	0	0	0	1	0	0	2	0	1	0
1396	3084621	2	0	1	5	1	1	1	0	0	1	0	0	0	0	1	0	0	2	0	1	0
1397	3084622	2	1	2	3	1	1	1	0	1	1	0	0	0	0	1	0	0	2	0	1	0
1398	3084623	2	1	2	4	1	1	1	0	1	1	0	0	0	0	1	0	0	2	0	1	0
1399	3084624	2	1	2	3	1	1	1	1	1	1	0	0	0	1	1	1	0	3	1	1	1
1400	3084625	2	0	2	4	1	1	1	0	1	1	0	0	0	0	1	0	0	2	0	1	0
1401	309471	2	1	4	4	2	1	1	1	1	1	1	1	1	1	1	1	1	4	1	1	1
1402	309472	2	0	3	3	1	1	1	1	1	1	1	1	0	1	0	1	1	4	1	1	1
1403	309473	2	1	2	3	1	1	1	1	0	1	1	0	0	1	1	1	1	3	1	1	1
1404	309474	2	1	2	4	1	1	1	1	0	1	1	0	0	1	1	1	1	3	1	1	1
1405	309475	2	0	2	2	1	1	1	1	0	1	0	1	1	1	1	1	1	4	1	1	1
1406	309476	2	0	4	3	3	1	1	1	0	1	1	1	1	1	1	1	1	4	1	1	1
1407	309477	2	1	2	2	2	1	1	1	1	1	0	1	0	1	0	1	1	3	1	1	1
1408	309478	2	1	4	4	1	1	1	1	1	1	1	1	1	1	1	1	1	4	1	1	1
1409	309479	2	1	2	5	1	1	1	1	0	1	1	1	0	1	0	1	1	3	1	1	1
1410	3094710	2	1	2	2	3	1	1	1	0	0	1	1	0	0	0	1	1	2	0	1	0
1411	3094711	2	0	2	2	1	1	1	1	1	1	1	1	0	1	0	1	1	4	1	1	1
1412	3094712	2	0	5	2	2	1	1	1	1	0	1	1	0	1	1	1	1	4	1	1	1
1413	3094713	2	1	2	1	2	1	1	1	0	0	0	0	0	0	0	0	0	2	0	1	0
1414	3094714	2	1	2	1	1	1	1	1	0	0	0	0	0	0	0	0	0	2	0	1	0
1415	3094715	2	1	2	4	1	1	1	1	0	0	0	1	0	1	1	1	1	3	1	1	0
1416	3094716	2	1	2	2	2	1	1	1	0	0	1	1	0	1	0	1	1	3	1	1	0
1417	3094717	2	1	5	2	3	1	1	1	1	1	1	1	0	1	1	1	1	4	1	1	1
1418	3094718	2	1	2	2	1	1	1	1	0	1	1	1	0	1	0	1	1	3	1	1	1
1419	3094719	2	0	3	2	2	1	1	1	1	0	1	1	0	1	0	1	1	3	1	1	1
1420	3094720	2	0	3	4	1	1	1	1	1	1	1	1	0	1	0	1	1	4	1	1	1
1421	3094721	2	1	2	3	1	1	1	1	1	1	1	1	0	1	0	1	1	4	1	1	1
1422	3094722	2	0	2	3	1	1	1	1	1	1	1	1	0	1	0	1	1	4	1	1	1
1423	3094723	2	1	3	3	1	1	1	1	1	1	1	1	0	1	0	1	1	4	1	1	1
1424	3094724	2	0	5	1	2	1	1	1	1	1	1	1	0	1	0	1	1	4	1	1	1
1425	3094725	2	1	2	5	1	1	1	1	0	1	1	1	0	0	0	1	1	3	1	1	0
1426	3094726	2	0	2	4	1	1	1	1	0	0	1	1	0	1	0	1	1	3	1	1	0
1427	3094727	2	1	3	3	1	1	1	1	1	1	1	1	0	1	0	1	1	4	1	1	1
1428	3094728	2	0	4	1	2	1	1	1	1	1	1	1	0	1	0	1	1	4	1	1	1
1429	3094729	2	0	4	3	1	1	1	1	1	1	0	1	0	1	0	1	1	3	1	1	1
1430	3094730	2	1	3	3	1	1	1	1	1	1	1	1	0	1	0	1	1	4	1	1	1
1431	3094731	2	0	2	3	1	1	1	1	1	1	1	1	0	1	0	1	1	4	1	1	1
1432	3094732	2	1	2	2	1	1	1	1	1	1	1	1	0	1	0	1	1	4	1	1	1
1433	3094733	2	0	2	2	2	1	1	1	1	1	1	1	0	1	0	1	1	4	1	1	1
1434	3094734	2	0	4	2	3	1	1	1	1	1	1	1	0	1	0	1	1	4	1	1	1
1435	3094735	2	0	4	3	1	1	1	1	0	0	1	1	0	1	0	1	1	3	1	1	0
1436	3094736	2	0	4	3	1	1	1	1	0	1	0	1	0	1	0	1	1	3	1	1	0
1437	3094737	2	0	3	4	2	1	1	1	0	1	1	1	0	1	0	1	1	3	1	1	1
1438	3094738	2	0	3	3	1	1	1	1	0	1	1	1	0	1	0	1	1	3	1	1	1
1439	3094739	2	0	2	3	1	1	1	1	0	0	1	1	0	1	0	1	1	3	1	1	0

1440	3094740	2	0	4	3	2	1	1	1	1	1	1	1	0	1	1	1	1	4	1	1	1
1441	309481	2	1	3	2	1	1	1	1	0	1	1	1	0	1	0	1	1	3	1	1	1
1442	309482	2	1	3	2	1	1	1	1	1	1	1	1	0	1	0	1	1	4	1	1	1
1443	309483	2	0	5	2	1	1	1	1	0	1	1	1	0	1	0	1	1	3	1	1	1
1444	309484	2	0	2	5	1	1	1	1	1	1	1	1	0	1	0	1	1	4	1	1	1
1445	309485	2	0	4	2	2	1	1	1	0	1	1	1	0	1	0	1	1	3	1	1	1
1446	309486	2	0	2	3	2	1	1	1	0	1	1	1	0	1	1	1	1	4	1	1	1
1447	309487	2	0	2	3	1	1	1	1	1	1	1	1	0	1	0	1	1	4	1	1	1
1448	309488	2	0	2	4	1	1	1	1	1	1	1	1	0	1	1	1	1	4	1	1	1
1449	309489	2	0	2	3	2	1	1	1	0	1	1	1	0	1	0	1	1	3	1	1	1
1450	3094810	2	0	2	3	1	1	1	1	1	1	1	1	0	1	0	1	1	4	1	1	1
1451	3094811	2	0	2	4	1	1	1	1	1	1	0	1	0	1	0	1	1	3	1	1	1
1452	3094812	2	1	2	5	1	1	1	1	1	0	1	1	0	1	0	1	1	3	1	1	1
1453	3094813	2	1	2	3	1	1	1	1	1	1	1	1	0	1	1	1	1	4	1	1	1
1454	3094814	2	1	2	2	2	1	1	1	1	1	1	1	0	1	0	1	1	4	1	1	1
1455	3094815	2	1	4	2	1	1	1	1	1	1	1	1	0	1	0	1	1	4	1	1	1
1456	3094816	2	0	2	2	1	1	1	1	1	1	1	1	0	1	0	1	1	4	1	1	1
1457	3094817	2	1	2	2	1	1	1	1	1	1	0	1	0	1	0	1	1	3	1	1	1
1458	3094818	2	1	2	1	1	1	1	1	0	1	1	0	0	0	0	1	0	2	0	1	0
1459	3094819	2	1	2	3	1	1	1	1	1	1	1	1	0	1	0	1	1	4	1	1	1
1460	3094820	2	0	2	2	1	1	1	1	0	1	1	1	0	1	0	1	1	3	1	1	1
1461	3094821	2	1	4	3	1	1	1	1	1	1	1	1	0	0	0	1	1	3	1	1	1
1462	3094822	2	1	2	5	1	1	1	1	0	1	1	1	0	1	0	1	1	3	1	1	1
1463	3094823	2	0	2	4	1	1	1	1	1	1	1	1	0	0	0	1	1	3	1	1	1
1464	3094824	2	1	2	5	1	1	1	1	0	1	0	1	0	1	0	1	1	3	1	1	0
1465	3094825	2	1	5	4	2	1	1	1	0	1	1	1	0	1	0	1	1	3	1	1	1
1466	309491	2	1	2	4	1	1	1	1	1	0	1	1	0	1	1	1	1	4	1	1	1
1467	309492	2	0	2	2	1	1	1	1	1	1	1	1	0	1	0	1	1	4	1	1	1
1468	309493	2	0	2	4	1	1	1	1	0	1	1	1	0	1	0	1	1	3	1	1	1
1469	309494	2	0	2	5	1	1	1	1	0	1	0	1	0	1	0	1	1	3	1	1	0
1470	309495	2	0	2	2	1	1	1	1	0	1	1	1	0	1	0	1	1	3	1	1	1
1471	309496	2	1	5	3	2	1	1	1	0	1	1	1	0	1	1	1	1	4	1	1	1
1472	309497	2	1	2	4	1	1	1	1	1	1	1	1	0	1	0	1	1	4	1	1	1
1473	309498	2	0	2	1	1	1	1	1	1	1	1	1	0	1	0	1	1	4	1	1	1
1474	309499	2	0	2	3	1	1	1	1	0	1	1	1	0	1	0	1	1	3	1	1	1
1475	3094910	2	1	3	3	2	1	1	1	0	1	1	1	0	1	0	1	1	3	1	1	1
1476	3094911	2	0	2	3	1	1	1	1	0	1	1	1	0	1	0	1	1	3	1	1	1
1477	3094912	2	0	5	2	2	1	1	1	0	1	1	1	0	1	0	1	1	3	1	1	1
1478	3094913	2	1	2	2	1	1	1	1	1	0	1	1	0	0	0	1	1	3	1	1	0
1479	3094914	2	1	3	2	1	1	1	1	1	1	1	1	0	1	1	1	1	4	1	1	1
1480	3094915	2	1	2	3	1	1	1	1	0	1	1	1	0	1	0	1	1	3	1	1	1
1481	3094916	2	1	2	4	1	1	1	1	1	0	1	1	0	1	1	1	1	4	1	1	1
1482	3094917	2	1	2	2	1	1	1	1	0	0	0	0	0	0	1	0	0	2	0	1	0
1483	3094918	2	0	4	2	1	1	1	1	0	1	1	1	0	1	1	1	1	4	1	1	1
1484	3094919	2	0	2	2	1	1	1	1	0	1	1	1	0	1	0	1	1	3	1	1	1
1485	3094920	2	0	2	3	2	1	1	1	0	1	1	1	0	1	0	1	1	3	1	1	1
1486	3094921	2	1	2	3	1	1	1	1	1	1	1	1	0	1	1	1	1	4	1	1	1
1487	3094922	2	0	2	3	1	1	1	1	1	1	1	1	0	1	0	1	1	4	1	1	1
1488	3094923	2	0	2	2	1	1	1	1	1	1	1	1	0	1	1	1	1	4	1	1	1
1489	3094924	2	0	5	1	3	1	1	1	1	1	1	1	0	1	0	1	1	4	1	1	1
1490	3094925	2	0	5	2	1	1	1	1	1	1	1	1	0	1	0	1	1	4	1	1	1
1491	3094926	2	1	4	4	2	1	1	1	1	1	1	1	0	0	1	1	1	4	1	1	1
1492	3094927	2	1	2	4	2	1	1	1	0	0	0	0	0	0	1	0	0	2	0	1	0
1493	3094928	2	1	5	2	2	1	1	1	1	1	1	0	0	1	1	1	1	4	1	1	1
1494	3094929	2	1	2	1	1	1	1	1	0	1	0	1	0	1	0	1	1	3	1	1	0
1495	3094930	2	1	2	3	1	1	1	1	1	1	1	1	0	1	0	1	1	4	1	1	1

Variables explanation

Res_ID	Respondent ID	
MES	Malaria endemic settings	
	1	High (East Sumba district)
	2	Low (East Manggarai district)
	3	Moderate (Belu district)
Sex	Gender of participants	
	0	Female
	1	Male
Edu	Education level of participants	
	1	No education
	2	Primary school
	3	Junior high school
	4	Senior high school'
	5	Diploma or above
AG	Age group of participants	
	1	< 30
	2	30 – 39
	3	40 – 49
	4	50 – 59
	5	> 60
SES	Social economic status of participants	
	1	Low
	2	Average
	3	Hig h
Q1	Hearing malaria term	
	0	No
	1	Yes
Q2	Dangerous effecf of malaria to health	
	0	No
	1	Yes
Q3	Awareness that malaria can be prevented	
	0	No
	1	Yes
Q4	The ability to identify fever as main sympthom of malaria	
	0	No
	1	Yes
Q5	The correct knowledge on mosquito bite as transmission mode of malaria	
	0	No
	1	Yes
Q6	Knowledge on sleeping under non-LLINs	
	0	No
	1	Yes
Q7	Knowledge on sleeping under LLINs	
	0	No
	1	Yes
Q8	Knowledge on using mosquito coil	
	0	No
	1	Yes
Q9	Knowledge on keep house clean	
	0	No
	1	Yes
Q10	Seeking treatment within 24 hours at health facility	
	0	No
	1	Yes
K_1	Know at least one prevention method	
	0	No
	1	Yes
K_2	Know at least two prevention method	
	0	No
	1	Yes

MKS	Malaria knowledge score	
	1	Very poor
	2	Poor
	3	Good
	4	Excellent
MA	Malaria awareness status of participants	
	0	Unaware
	1	Aware
ABUM	Awareness of basic malaria understanding of participants	
	0	No
	1	Yes
ABMK	Awareness of basic malaria knowledge	
	0	No
	1	Yes
