# Supplementary material for: Malaria awareness of adults in high, moderate and low transmission settings: A cross-sectional study in rural East Nusa Tenggara Province, Indonesia
Source: PLoS One. 2021 Nov 15;16(11):e0259950. doi: 10.1371/journal.pone.0259950 (PMC8592438; doi:10.1371/journal.pone.0259950)
Supplement: Attachment — Submitted filename: Response to Reviewers.docx [file pone.0259950.s003.docx]

**rebuttal letter**

PONE-D-20-34159
Malaria awareness of adults in high, moderate and low transmission settings: A cross-sectional study in rural East Nusa Tenggara Province, Indonesia

Journal Requirements:

1. Please ensure that your manuscript meets PLOS ONE's style requirements, including those for file naming. The PLOS ONE style templates can be found at https://journals.plos.org/plosone/s/file?id=wjVg/PLOSOne_formatting_sample_main_body.pdf and [https://journals.plos.org/plosone/s/file?id=ba62/PLOSOne_formatting_sample_title_authors_affiliations.pdf](about:blank)

**Response:**

Thank you for this feedback. We have updated the manuscript to meet the PLOS ONE style requirements including file naming.

2. Please include a copy of the questionnaire in the original language as Supporting Information or include a citation if it has been published previously.

**Response:**

The original language of the questionnaire is a part of published protocol paper. We have cited this protocol paper in our manuscript as reference number 33.

3. In the Methods, please discuss whether and how the questionnaire was validated and/or pre-tested. If these did not occur, please provide the rationale for not doing so.

**Response:**

Thank you for this recommendation. The questionnaire used for this study has been adapted from a validated questionnaire which was published previously (reference numbers 35 and 36). The questionnaire was translated into local language and we also conducted a pre-test upon 30 participants before finalising the questionnaire. This is now explained in the data collection section under material and methods (page 8 line 2 – 13).

4. In your statistical analyses, please state whether you accounted for clustering by region. For example, did you consider using multilevel models?

**Response:**

In this study, we want to develop malaria awareness of rural ENTP and evaluate the association between malaria awareness and three malaria endemic settings (MES). Since the main question for this study is whether any a significant association between malaria awareness having binary response and different types of MES, we believe the logistic regression method is suitable for this research question. Furthermore, we do not want to predict malaria awareness amongst three different MES based on their characteristics, therefore we did not use multilevel models for this study. Reporting the malaria awareness as a whole was not our objective.

5. Please include your tables as part of your main manuscript and remove the individual files. Please note that supplementary tables should remain uploaded as separate "supporting information" files.

**Response:**

We have now included Table 1 and Table 2 in this manuscript. However, the size of Table 3 was big. Following the guideline of PLOS One Journal, if the size of table was not fitted in the manuscript due to the its size, it can be transformed to figure and uploaded as separate file. Therefore, we uploaded Table 3 as a figure in a different file.

6. We note that you have stated that you will provide repository information for your data at acceptance. Should your manuscript be accepted for publication, we will hold it until you provide the relevant accession numbers or DOIs necessary to access your data. If you wish to make changes to your Data Availability statement, please describe these changes in your cover letter and we will update your Data Availability statement to reflect the information you provide.

**Response:**

We have attached our database in this manuscript to support our findings as supporting information in this submission as shown in page 23 line 24.

7. We note that Figure 1 in your submission contain map images which may be copyrighted. All PLOS content is published under the Creative Commons Attribution License (CC BY 4.0), which means that the manuscript, images, and Supporting Information files will be freely available online, and any third party is permitted to access, download, copy, distribute, and use these materials in any way, even commercially, with proper attribution. For these reasons, we cannot publish previously copyrighted maps or satellite images created using proprietary data, such as Google software (Google Maps, Street View, and Earth).

We require you to either (1) present written permission from the copyright holder to publish these figures specifically under the CC BY 4.0 license, or (2) remove the figures from your submission:

**Response:**

Thank you the academic editor for this recommendation. We have updated the figure 1 for the location of the study. The map was developed by the author of this article. We developed map using ggplot2 and map package in R software version 3.5.3

8. Please include captions for your Supporting Information files at the end of your manuscript, and update any in-text citations to match accordingly. Please see our Supporting Information guidelines for more information: [http://journals.plos.org/plosone/s/supporting-information](about:blank).

**Response:**

We have added in-text citations for this supporting information as indicated in page 6 line 23, page 16 line 9, and page 17 line 10. The caption for the supporting information files has been also added at the end of our manuscript as shown in page 23 line 19 and 24.

**Reviewer's Responses to Questions**

5. Review Comments to the Author

**Reviewer #1**: In the introduction section, the author should mention and analyze several strategies that have been conducted in the ENTP to eliminate malaria. This explanation would give a more substantial argument why study regarding malaria knowledge related to the symptom, transmission mode, prevention method, and the perception of malaria treatment-seeking behavior in the population-level study, thus important.

**Response:**

We have updated the introduction section of the manuscript. We have presented some intervention methods that have been implemented in the ENTP as a part of the effort of the local government to eliminate malaria by 2030. However, their intervention might be ineffective since the number of malaria cases is still high and their intervention might be dependent on the knowledge and behaviour of the local community which is limited investigated in the study area as indicated in page 4 from line 11 to 25 and page 5 from line 1 to 4. We have also shown the gap between previous malaria studies on malaria knowledge in the ENTP and the current study that we have conducted in order to provide the best solution for malaria elimination program in the ENTP as shown in page 5 from line 18 to 25 and page 6 from line 1 to 11.

**Reviewer #2:** abstract
Materials and methods
study population, sample size and sampling method were not indicated.

**Response:**

We have updated the material and methods section in the abstract. The information on the study population, sample size and sampling method has been added in the abstract as indicated page 2 from line 8 to 11.

Strong model like logistic regression is preferable to chi square

**Response:**

Thank you to the reviewer #2 for this suggestion. The purpose of this article is to develop malaria awareness index and to evaluate the association of the malaria awareness index amongst community in three different malaria endemic settings of rural adults in ENTP Indonesia. The outcome variables is proportion of rural adults having malaria awareness in each different types of malaria endemic settings. Firstly, we show the association between malaria endemic settings and basic malaria understanding, basic malaria knowledge, as well as malaria awareness applying chi-square test. Furthermore using odds ratio in logistic regression model, we have shown that there is a significant association between malaria endemic settings and basic malaria understanding, basic malaria knowledge, as well as malaria awareness. This information has been updated in the article as shown in page 2 line 12 and 13, page 10 line 19 to 25 and page 11 from line 1 to 3.

**Results**

The proportion of malaria awareness index should be specified along with CI.

**Response:**

Thank you to the review #2 for this recommendation. We have updated the result section of the abstract. The proportion of malaria awareness index has been specified along with 95% confidence interval as indicated in page 2 from line 16 to 25 and page 3 line 1.

**Main body**
**Introduction**

Research gap was not indicated

**Response:**

Thank you to the reviewer #2 to this recommendation. We have updated the manuscript. We have updated 2 paragraphs in the manuscript to indicate the research gap in our paper. For the first paragraph, as indicated in page 4 from line 11 to 25 and page 5 from line 1 to 4 of the manuscript, we have presented some evidence of interventions conducted by local authority and independent researchers as a part of their effort to eliminate malaria in line with the national commitment of the country. Most of their intervention might be ineffective since the number of malaria cases still high in the province and the implementation of their intervention might depend on the community behaviour which is limited investigated in the study area. So this is the main reason for conducting this study.

For the second paragraph of the research gap, as indicated in page 5 from line 18 to 25 and page 6 from line 1 to 11 of the manuscript, we have updated the article with the new evidence on malaria awareness research in the ENTP indicating low awareness of community in malaria prevention method using the long-lasting insecticide- treated nets (LLINs), however their study evaluated only awareness of pregnant women on malaria prevention method living in high malaria endemic settings (MES). Therefore, their study cannot compare the malaria awareness amongst other types of MES in the ENTP. In our study, we develop malaria awareness of rural adults in ENTP and investigate the difference of malaria awareness amongst different types of MES. These findings allow us to provide new evidence which MES should be prioritized and which aspect should be focus in providing key intervention for rural community in the effort to progress to malaria elimination by 2030. Therefore, our study will provide the novelty of malaria awareness of rural ENTP and the comparison of malaria awareness amongst MES in the ENTP.

**Materials and methods**
Sample size was not determined.

**Response:**

Thank you to the reviewer #2 for this suggestion. We have updated the article. Overall procedure on how to calculate the sample size has been added in the article. We determined our sample size taking into account of malaria prevalence study previously, design effect, cluster size, and participation rate of the participants in East Nusa Tenggara Province Indonesia. The comprehensive information on the calculation of sample size has been published in our prior publication as indicated in the reference number 33 of this manuscript. All this information were presented in page 7 from line 3 to 11.

Data collection method was specified

**Response:**

We have updated the article and data collection method has been added in this manuscript. For this study we applied face-to-face interview guided by the validated questionnaire as indicated in page 8 from line 3 to 13.

Malaria awareness assessment was not clear.

**Response:**

Thank you to the reviewer #2 for this recommendation. We have updated the article. We have presented ten questions used to evaluate malaria awareness of participants. The first three questions categorized as basic understanding of malaria including whether participants have heard malaria term, whether malaria was dangerous for their health, whether malaria can be prevented. The next seven questions categorized as basic malaria knowledge comprising whether participants could identify the main symptom and the main cause of malaria, whether participants could identify some protective measure to prevent malaria, whether participants seeking treatment for their malaria within 24 hours after the onset of the symptoms. Participants who could identify fever as the main symptom of malaria and mosquito bites as the main cause of malaria obtained score one respectively. Participants who could mention sleeping under non-LLINs, sleeping under LLINs, using mosquito coils, keeping house clean as the method to prevent malaria got score one respectively. Finally, participants who mention seeking malaria treatment within 24 hours for their malaria obtained score one.

Overall, each participants get a total score of ten if they could answer correctly all these questions. Total marks of ten questions were evaluated following the previous malaria awareness studies as indicated in the reference number 37 and 38. Participants answering correctly at least 60% for these ten questions were categorized as having malaria awareness. The classification of participants to be categorized as aware or unaware of basic malaria understanding and basic malaria knowledge was also following the guidance of previous study (reference number 37 and 38). All this explanation have been updated in the article as shown in page 8 from line 16 to 24 and page 9 from line 1 to 22.

.

Confidentiality issue and privacy was stated.

**Response:**

In the ethic approval section of this article, we have presented that this study has been approved by ethics committee of Swinburne University of Technology and Health Ministry of Indonesia government where we extensively addressed how we would address issues around confidentiality. The confidential issue and privacy of participants has also followed the procedure in the tenet of The Declaration of Helsinki as indicated in page 11 from line 6 to 16 in the manuscript. The evidence of ethics approval from Swinburne University of Technology and Health Ministry of Indonesia government has been uploaded to the system.

**Results**

The proportion of malaria awareness index should be specified along with CI.

**Response:**

Thank you to the reviewer #2 for this recommendation. We have updated the article and 95% of confidence interval of the proportion of malaria awareness index has been calculated and added in this manuscript as indicated in page 13 from line 10 to 19 and from page 14 to 17. The proportion of malaria awareness index in Table 2 has been also supported with 95% confidence interval as indicated in page 15.

The findings were not written in logical order

**Response:**

Thank you to the reviewer #2 for this recommendation. We have updated the article. We arranged to present our findings into four themes including demographic characteristic of respondents, malaria knowledge of participants, malaria awareness of participants, the strength of the association between malaria awareness and malaria endemic settings (MES). In each theme, we compared the interest point amongst MES.

The demographic characteristic of participant was presented in page 11 from line 20 to 24 and page 12 from line 1 to 3. Malaria knowledge of participants was divided into basic malaria understanding as indicated in page 13 from line 7 to line 13, and the basic malaria knowledge as shown in page 15 from line 19 to line 16 and page 14 from line 1 to 2. Knowledge on malaria prevention methods was presented in page 14 from line 4 to 15, and the understanding of treatment seeking behaviour was shown in page 14 from line 17 to 22. In each section, we made comparison of understanding amongst three different malaria endemic settings.

The main result, malaria awareness index of participants was presented at page 16. In this part, we started with the awareness of basic malaria understanding and basic malaria knowledge as indicated in page 16 from line 14 to 22. Next, we presented malaria awareness index of participants as shown in page 16 from line 24 to 25 and page 17 from line 1 to 4. In each part, the comparison of malaria awareness amongst three different types of malaria endemic settings has been provided.

Finally, at the last part of the result section, we presented the strength of association between malaria awareness index and MES as shown in page 17 from line 10 to 18.

**Discussions**
The findings were not well written.

**Response:**

Thank you to the reviewer #2 for this recommendation. We have improved the manuscript. Firstly, we present our main findings which are the low level of malaria awareness amongst rural community in the ENTP and the high MES has the lowest malaria awareness as indicated in page 17 from line 21 to 24 and page 18 from line 1 to 2.

Then we compare our main findings with other countries and we provided explanation on why there is a significant discrepancy in malaria awareness in ENTP Indonesia and other countries that had implemented malaria intervention to improve malaria knowledge of the rural community as indicated in page18 from line 2 to 18.

The next part is presenting the discussion on malaria awareness in more detail started from basic malaria knowledge on main symptom and main causes as indicated in page 18 from line 20 to 25 and page 19 from line 1 to 9. The discussion on the awareness on sleeping under LLINs to prevent malaria and the comparison with other countries as well as the reason for the difference in ENTP and other countries has been presented in page 19 from line 1 to 25 and page 20 from line 1 to 7.

The discussion on awareness on malaria prevention method and the possibility reason why there is a discrepancy in this awareness between MES in ENTP was provided in the page 20 from line 9 to 19.

Regarding to malaria treatment seeking behaviour and the discussion of why the level of this awareness was low, it is presented in page 20 from line 21 to 25 and page 21 from line 1 to 6.

The next part we provided some recommendations to support malaria elimination program in the ENTP as indicated in page 21 from line 8 to 25 and page 22 from line 1 to 4.

Finally the strength and limitation of the research has been presented at the end of the discussion as indicated in page 22 from line 6 to 17.

The findings have not been contrasted.

**Response:**

Thank you to the reviewer #2 for this recommendation. We have improved the manuscript. We have compared our findings with malaria studies in other countries. The main findings of the study was the low level of malaria awareness of rural population and the lowest malaria awareness was in high Malaria endemic setting (MES) in ENTP Indonesia. This finding has been compared with 6 studies in different countries and we provided an argument to explain the possibility reason for the discrepancy of malaria awareness between ENTP Indonesia and other countries as shown in page 18 from line 4 to 18.

The awareness of various aspects of malaria has been also compared with another countries. The awareness of basic malaria knowledge on main malaria symptom has been compared with 2 studies in different countries as indicated in page 18 from line 20 to 25. The awareness on the main causes of malaria has been compared with similar study in 5 countries as shown in page 19 from 1 to 9.

The awareness of sleeping under LLINs to prevent malaria has been also compared with similar studies in 6 countries. The possibility reason for the disparity of this awareness in ENTP Indonesia and other countries has been also provided as indicated in page 19 from line 20 to 25 and page 20 from line 1 to 7. Meanwhile, for the difference in the awareness of sleeping under LLINs amongst MES in ENTP has been compared with similar studies in 2 countries. The possibility reason for the trend has also been supported as indicated in page 20 from line 9 to 19.

Finally, the awareness of treatment seeking behaviour of rural population ENTP Indonesia has been compared with similar studies in 2 other provinces of Indonesia and in 4 countries in Asia. The discussion on this low awareness has been also provided as indicated in page 20 from line 21 to 25 and page 21 from line 1 to 6.
