# Supplementary material for: Malaria awareness of adults in high, moderate and low transmission settings: A cross-sectional study in rural East Nusa Tenggara Province, Indonesia
Source: PLoS One. 2021 Nov 15;16(11):e0259950. doi: 10.1371/journal.pone.0259950 (PMC8592438; doi:10.1371/journal.pone.0259950)
Supplement: Attachment — Submitted filename: Response to Reviewers.docx [file pone.0259950.s004.docx]

**rebuttal letter**

PONE-D-20-34159R1
**“Malaria awareness of adults in high, moderate and low transmission settings: A cross-sectional study in rural East Nusa Tenggara Province, Indonesia”**
PLOS ONE

Journal Requirements:

Please review your reference list to ensure that it is complete and correct. If you have cited papers that have been retracted, please include the rationale for doing so in the manuscript text, or remove these references and replace them with relevant current references. Any changes to the reference list should be mentioned in the rebuttal letter that accompanies your revised manuscript. If you need to cite a retracted article, indicate the article’s retracted status in the References list and also include a citation and full reference for the retraction notice.

**Response:**

Thank you for this feedback. We have updated the reference list. Since the Indonesian government has released the current number of malaria cases in Indonesia, we have changed the reference [8] to the year 2020. We have updated all this information as indicated on page 4 in the manuscript. All other references have been prepared following the guidance of the journal.

Reviewers' comments:

Reviewer's Responses to Questions

**Comments to the Author**

1. If the authors have adequately addressed your comments raised in a previous round of review and you feel that this manuscript is now acceptable for publication, you may indicate that here to bypass the “Comments to the Author” section, enter your conflict of interest statement in the “Confidential to Editor” section, and submit your "Accept" recommendation.

Reviewer #1: All comments have been addressed

Reviewer #2: (No Response)

**Response:**

Thank you for your comments

2. Is the manuscript technically sound, and do the data support the conclusions?

   The manuscript must describe a technically sound piece of scientific research with data that supports the conclusions. Experiments must have been conducted rigorously, with appropriate controls, replication, and sample sizes. The conclusions must be drawn appropriately based on the data presented.

Reviewer #1: Yes

Reviewer #2: Yes

**Response:**

Thank you for your comments

3. Has the statistical analysis been performed appropriately and rigorously?

Reviewer #1: Yes

Reviewer #2: Yes

**Response:**

Thank you for your comments

4. Have the authors made all data underlying the findings in their manuscript fully available?

   The [PLOS Data policy](http://www.plosone.org/static/policies.action#sharing) requires authors to make all data underlying the findings described in their manuscript fully available without restriction, with rare exception (please refer to the Data Availability Statement in the manuscript PDF file). The data should be provided as part of the manuscript or its supporting information, or deposited to a public repository. For example, in addition to summary statistics, the data points behind means, medians and variance measures should be available. If there are restrictions on publicly sharing data—e.g. participant privacy or use of data from a third party—those must be specified.

Reviewer #1: Yes

Reviewer #2: No

**Response:**

All data underlying the findings of this manuscript has been made available as a part of supporting information of this manuscript.

5. Is the manuscript presented in an intelligible fashion and written in Standard English?

   PLOS ONE does not copyedit accepted manuscripts, so the language in submitted articles must be clear, correct, and unambiguous. Any typographical or grammatical errors should be corrected at revision, so please note any specific errors here.

Reviewer #1: Yes

Reviewer #2: No

**Response:**

We have sought the assistance of a professional editor to review our paper to address these concerns (a certificate is attached). All authors have spent considerable time editing this paper.

6. Review Comments to the Author

Please use the space provided to explain your answers to the questions above. You may also include additional comments for the author, including concerns about dual publication, research ethics, or publication ethics. (Please upload your review as an attachment if it exceeds 20,000 characters)

Reviewer #1: Dear Author,
I appreciate your hard work improving this paper and appreciate your responsiveness.
Your response to my comments and your revision according to the comments are acceptable.
You did a good job.
Good luck.

Reviewer #2: Major assumptions of logistics regression analysis like Multi collinearity not stated. Model fitness test was not indicated. What was outcome measure? What is the type of test? The results of logistics regression analyses were not presented.

**Response:**

Thank you to the reviewer #2 to this recommendation.

For the assumption of multi-collinearity, since there is a significant correlation between education level and socio-economic status (SES), we removed the SES variable from the logistic regression model for covariate adjustment. With or without SES also did not change the Wald statistics significantly. Therefore, the final odds ratio was obtained after adjusted for gender, age group and education level.

There are three outcome measures of the study. They are basic malaria understanding (the first outcome), basic malaria knowledge (the second outcome), and malaria awareness (the third outcome). All these outcomes were binary variables as indicated on page 8 from line 2 to 25 and page 9 from line 1 to 7.

The overall model fit test was evaluated by the omnibus chi-square test and classification tables. The value of the omnibus chi-square test was significant for all outcomes of the study (p-value < 0.001). This indicates an association between outcome variables and malaria-endemic settings. The classification tables also indicate the significant results for three outcomes. The classification accuracy for the first, second and third outcomes was 83.6%, 69.4%, and 66.3%, respectively.

For the type of test, the significance of the individual variable was evaluated by Wald statistics. All Wald statistics for three outcomes show a significant result (p-value < 0.001).

The results of logistic regression have been presented in figure 3, as indicated on page 16 of the manuscript.
