# Supplementary material for: Malaria awareness of adults in high, moderate and low transmission settings: A cross-sectional study in rural East Nusa Tenggara Province, Indonesia
Source: PLoS One. 2021 Nov 15;16(11):e0259950. doi: 10.1371/journal.pone.0259950 (PMC8592438; doi:10.1371/journal.pone.0259950)
Supplement: Attachment — Submitted filename: Response to Reviewers.docx [file pone.0259950.s005.docx]

**rebuttal letter**

PONE-D-20-34159R2
**Malaria awareness of adults in high, moderate and low transmission settings: A cross-sectional study in rural East Nusa Tenggara Province, Indonesia**
PLOS ONE


Journal Requirements:

Please review your reference list to ensure that it is complete and correct. If you have cited papers that have been retracted, please include the rationale for doing so in the manuscript text, or remove these references and replace them with relevant current references. Any changes to the reference list should be mentioned in the rebuttal letter that accompanies your revised manuscript. If you need to cite a retracted article, indicate the article’s retracted status in the References list and also include a citation and full reference for the retraction notice.

**Response:**

Thank you for this feedback. We have updated the reference list. We added reference [31] to support the sample size calculation, reference [43] for controlling confounding factors, and reference [53], [54], [55], [56] to support the argument for the association between malaria awareness and education level in the discussion section. All references have been prepared following the guidance of the journal.

**Reviewers' comments:**

**Reviewer #2: Abstract**

The authors did not write their manuscript rigorously.
The following concerns should be addressed:

1. Types of study, data collection technique, and types of logistic regression analysis were not specified.

**Response:**

We have updated methods section in the abstract as:

“A community-based cross-sectional study was conducted between October and December 2019 in high, moderate, and low malaria-endemic settings (MESs) in the ENTP. After obtaining informed consent, data were collected using an interviewer-administered structure questionnaire among 1503 participants recruited by a multi-stage cluster sampling method. A malaria awareness index was developed based on ten questions. A binary logistic regression method was applied to investigate the significance of any association between malaria awareness and the different MESs” (page 2, lines 8 -14).

**Main body**
**Material and methods**

There was major methodological defect:

1. types of study, Source population, study population, inclusion criteria and exclusion criteria were not stated

**Response:**

We have updated the material and method section. The study site is updated by including

“This community-based cross-sectional study was conducted from October to December 2019 in three districts out of 21 districts and one municipality in the province. They were East Sumba, Belu, and East Manggarai districts representing high, moderate, and low MESs, respectively” (page 6, lines 17-20).

The sampling technique section is updated by including

“All adults in ENTP were the source population, and all adults in the selected three districts were the study population” (page 7, lines 14 -15).

“In each selected household, one head of the family of any gender who provided consent to participate voluntarily was included in the study. If the household head, either husband or wife, was absent, residents over 18 years of age could serve as study participants [33]. We excluded anyone under the age of 18 years old from the study” (page 7, lines 22 - 25).

2. Sample size determination was not succinctly computed.
   Design effect was not considered.

**Response:**

Thank you for this feedback. We have updated the sample size determination section as below:

“The initial sample size (n_0_) was calculated based on the formula n_0_ = Z^2^P(1-P)/d^2^ for the prevalence study of a cross-sectional study [31]. The parameters Z is the value of standard score of 95% confidence interval (1.96), P is the prevalence of malaria study in the ENTP conducted by the Indonesian government (1.99%) [9] , and d is the relative precision which is 0.01125. Therefore, the initial sample size n_0_ was equal to 592. The design effect was accounted for due to cluster sampling by the multiplication of a factor of 2.16. By considering the participation rate of 85%, the final sample size was 1503 adults. The sample size calculation was described previously [32]” (page 7, lines 3-10).

3. Sampling technique was not clearly stated

**Response:**

Thank you for this feedback. We have updated the sampling technique section as below:

**“**A multi-stage cluster sampling procedure with a systematic random sampling procedure at the final cluster level was applied to recruit adults from the three districts. At cluster level 1, three districts were selected out of 22 in the ENTP based on the annual parasite incidence (API) of malaria, at cluster level 2, three sub-districts were randomly chosen from each selected district. At cluster level 3, the number of villages selected from each sub-district was based on their relative populations. At the final cluster level, a systematic random sampling technique was used to recruit 20–40 participants per village, proportionate to the population size of each village” (page 7, lines 15 – 22).

4. Data collection technique

**Response:**

Thank you for this feedback. We have improved data collection technique section by including the following information:

“An interviewer-administered questionnaire adapted from a validated questionnaire [34, 35] was used to collect data for this study. The English version of the questionnaire was translated into the local language by the lead author of this article and a local language expert. They then combined the two translated versions. The combined version of the questionnaire was then tested on 30 participants before finalization. The data were collected in collaboration with local nurses who were residents in the study area. Nine local nurses, three nurses from each district conducted face-to-face interviews with participants based on the guidance of the structured questionnaire. The data collection process was monitored strictly by the investigator daily to check the questionnaire's completeness. Data on the socio-demographic variables and general knowledge of malaria was collected during the interview” (page 8, lines 2 – 11).

5. Descriptive statistics, and types of logistic regression analysis were not specified, model assumptions (multicollinearity) was not checked and its value was not indicated. Was there interaction?

**Response:**

Thank you for this feedback. The descriptive statistics has been specified in the statistical analysis section as:

“The proportion of participants answering each question correctly and its 95% confidence interval (CI) were computed for each MES” (page 10, lines 17-19).

Types of logistic regression, adjustment and addressing the issue of multicollinearity have been presented in Statistical analysis section as:

“A univariate and multivariate binary logistic regression model was applied to evaluate the association between the dependent and the independent variables. The associations were reported as odds ratio with its 95% CI. Multicollinearity tests amongst the independent variables were done before multivariate analysis was conducted” (page 10, lines 22 – 25 and page 11 line 1).

6. How did you estimate strength of association b/n explanatory variables and endpoint?

**Response:**

Thank you for this feedback. The strength of association between explanatory variables and endpoint has been specified in the statistical analysis section as:

“The direction and strength of association between explanatory variables and endpoints were estimated by adjusting the odds ratio” (page 11, lines 7 – 8).

7. What are the strategies to CONTROL CONFOUNDERS?

**Response:**

Thank you for this feedback. Strategies to control confounders have been specified in the statistical analysis section as:

“In the univariate binary logistic regression, all variables having a p-value < 0.10 were included in the multivariate analysis to control confounding factors [43]” (page 11, lines 3 – 5).

8. How did you identify model fitness?

**Response:**

Thank you for this feedback. The model fitness test has been specified in the statistical analysis section as:

“The Hosman and Lemeshow test evaluated the overall model fitness with a significance level of p < 0.05” (page 11, lines 1-2).
